# Long-term super-resolution inner mitochondrial membrane imaging with a lipid probe

**DOI:** 10.1038/s41589-023-01450-y

**Published:** 2023-10-19

**Authors:** Shuai Zheng, Neville Dadina, Deepto Mozumdar, Lauren Lesiak, Kayli N. Martinez, Evan W. Miller, Alanna Schepartz

**Affiliations:** 1 Department of Chemistry, University of California, Berkeley, Berkeley, CA USA; 2 Department of Chemistry, Yale University, New Haven, CT USA; 3 Department of Molecular and Cell Biology, University of California, Berkeley, Berkeley, CA USA; 4 California Institute for Quantitative Biosciences, University of California, Berkeley, Berkeley, CA USA; 5 Chan Zuckerberg Biohub, San Francisco, San Francisco, CA USA

**Keywords:** Chemical tools, Cell biology, Membranes

## Abstract

The inner mitochondrial membrane (IMM) generates power to drive cell function, and its dynamics control mitochondrial health and cellular homeostasis. Here, we describe the cell-permeant, lipid-like small molecule MAO-N_3_ and use it to assemble high-density environmentally sensitive (HIDE) probes that selectively label and image the IMM in live cells and multiple cell states. MAO-N_3_ pairs with strain-promoted azide–alkyne click chemistry-reactive fluorophores to support HIDE imaging using confocal, structured illumination, single-molecule localization and stimulated emission depletion microscopy, all with significantly improved resistance to photobleaching. These probes generate images with excellent spatial and temporal resolution, require no genetic manipulations, are non-toxic in model cell lines and primary cardiomyocytes (even under conditions that amplify the effects of mitochondrial toxins) and can visualize mitochondrial dynamics for 12.5 h. This probe will enable comprehensive studies of IMM dynamics with high temporal and spatial resolution.

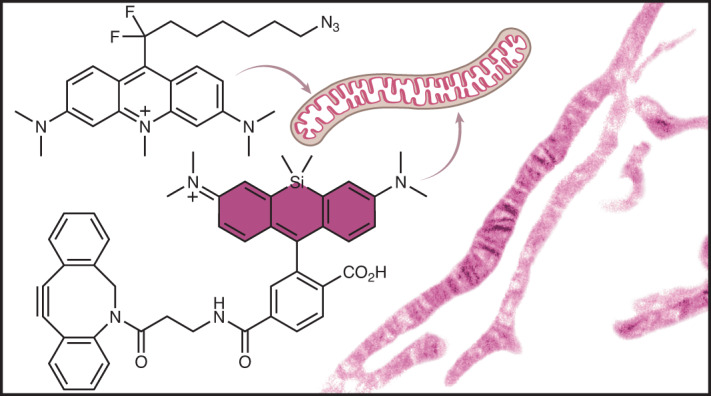

## Abstract (web summary)

A lipid-like small molecule, MAO-N_3_, was developed to assemble inner mitochondrial membrane-specific probes for confocal and various super-resolution microscopy techniques, with significantly improved time-lapse capability and minimal toxicity.

## Main

The mitochondrion is the powerhouse of the cell and contributes to numerous physiological processes, including programmed cell death, innate immunity, autophagy, redox signaling, calcium homeostasis and stem cell reprogramming. The mitochondrion powers the cell using oxidative phosphorylation, sustains the cell via multiple signaling activities and kills the cell when necessary by initiating programmed cell death^1^. Mitochondria are distinguished by multiple membrane structures that each perform distinct functions. The outer mitochondrial membrane (OMM) acts as a diffusion barrier, transduces signals in and out of the intermembrane space and mediates multiple interorganelle interactions^2^. The inner mitochondrial membrane (IMM) consists of two discrete elements, the inner boundary membrane and the cristae. The inner boundary membrane parallels the OMM and contains transporters that shuttle ions and metabolites between the intermembrane space and the mitochondrial matrix, which houses enzymes responsible for the tricarboxylic acid cycle and heme biosynthesis. The cristae are densely packed membrane invaginations with diffraction-limited dimensions that support oxidative phosphorylation, mitochondrial DNA maintenance and iron–sulfur cluster biogenesis^1^. The IMM and OMM undergo continual fission and fusion, and individual cristae remodel continuously in response to signals from other organelles^2^. These dynamic events drive mitochondrial biogenesis and recycling and play a pivotal role in apoptosis signaling^2^. Multiple pathological conditions in both neurons (autosomal-dominant optic atrophy)^2,3^ and energy-demanding myocytes (Barth’s syndrome^4,5^ and Kearns–Sayre syndrome^6^) are associated with mutations in IMM-localized enzymes. The on-demand initiation of mitochondrial apoptosis is also desirable in the development of novel cancer therapies^7^.

Imaging the IMM is challenging due to its complex and diffraction-limited dimensions, especially if the goal is to study dynamics. Respiratory chain enzymes within cristae are sensitive to fluorophore-mediated or high-energy light-induced damage^8,9^, which complicates the use of super-resolution methods, such as stimulated emission depletion (STED) microscopy^10^ and single-molecule localization microscopy (SMLM)^11,12^. Some of these challenges are alleviated using small-molecule dyes to image IMM dynamics at super-resolution. MitoPB Yellow, for example, is relatively photostable, localizes selectively to the IMM and can be combined with STED microscopy to image IMM dynamics for extended periods of time (300 frames over 390 s)^13^. However, MitoPB Yellow has limited spectral tuning potential for multicolor imaging, and longer imaging times are precluded by the requisite high-energy (488 nm) excitation wavelength (mitochondrial swelling is induced after as few as 150 frames). Moreover, because MitoPB Yellow is not modular, it is not easily adapted to other imaging modalities. MitoESq-635 (ref. ^9^) and PK Mito Orange (PKMO)^14^ also localize selectively to the IMM and are sufficiently photostable for STED imaging. MitoESq-635 is excited by lower-energy light (633 nm) than MitoPB Yellow but is also less photostable, supporting the acquisition of only 40 STED frames over 50 min to visualize IMM dynamics^9^. PKMO is also excited by lower-energy light (591 nm) and supports two-color STED imaging but can support only 33 frames over 5 min (ref. ^14^). These comparisons emphasize the need for new tools that support long time-lapse imaging of the mitochondrial inner membrane with high spatiotemporal resolution. The ideal tools to study mitochondrial cristae remodeling and interorganelle cross-talk should be bright, photostable, spectrally tunable, capable of supporting multiple imaging modalities and derived from small molecules to enable studies in primary cell lines.

**Fig. 1:**
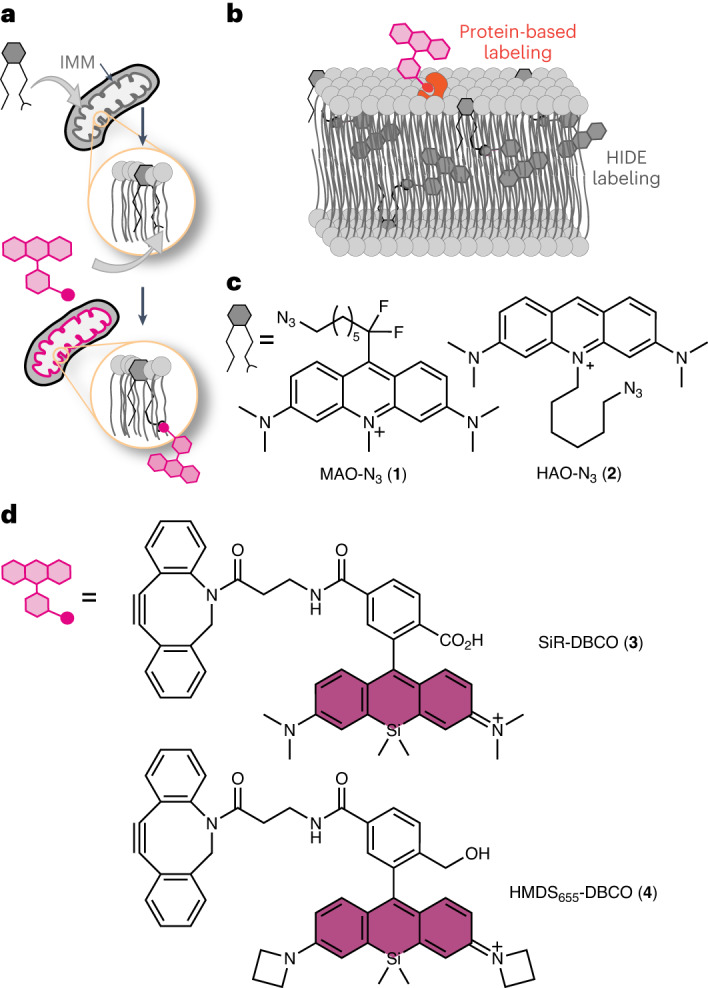
New HIDE probes for imaging the IMM. **a**, A HIDE probe is assembled in cellulo from two cell-permeant components. One component is a lipid-like small molecule appended to a reactive bioorthogonal functional group, such as N_3_. The second component is a modality-specific fluorophore equipped with the appropriate click reaction partner. **b**, HIDE probes extend imaging time because they localize within organelle membranes at a higher density than possible with a self-labeling or fluorescent protein and because, in this hydrophobic environment, only a small fraction of the molecules absorb light at the excitation wavelength. Because fewer molecules absorb light at any given time, fewer molecules bleach; what remains constitutes a reservoir of fresh fluorophores that replenishes photobleached dye molecules during an imaging experiment. **c**, Derivatives of acridine orange, namely MAO-N_3_ and HAO-N_3_, used to generate the HIDE probes reported here. **d**, Structures of two silicon rhodamine dyes used in conjunction with MAO-N_3_ and HAO-N_3_. HMDS_655_-DBCO was used for SMLM, whereas SiR-DBCO was used for all other imaging modalities.

High-density environmentally sensitive (HIDE) probes are small molecules that selectively label organelle membranes and resist photobleaching, leading to exceptionally long time-lapse images of organelles in live cells, even at super-resolution^15–22^. Each HIDE probe consists of two parts (Fig. 1a). The first part is an organelle-specific lipid or lipid-like small molecule that is click ready by virtue of a reactive functional group such as an azide (N_3_). The second part is a far-red fluorophore equipped with the appropriate reaction partner. These two parts, when added sequentially to live cells, undergo a bioorthogonal reaction that localizes the fluorophore at high density within the organelle membrane (Fig. 1b)^18^. The combination of dense localization and a hydrophobic environment results in a 10- to 50-fold enhancement in apparent photostability. HIDE probes have been used for long time-lapse imaging of the endoplasmic reticulum (ER), Golgi, plasma membrane, mitochondrial matrix and endolysosomes^15–22^. HIDE probes label organelles in model cell lines, primary cells from human samples^20^ and even neurons^19,21^. Because HIDE probes are assembled from two parts, the emission properties can be altered at will to support multicolor labeling and all established modalities^21^. One mitochondrial HIDE probe (RhoB-TCO) has been reported^18,21^, but it labels mitochondrial subcompartments with minimal specificity, is not selective for the IMM and cannot resolve IMM dynamics and fine structure^18,21^.

Here, we describe two lipid-like small molecules, *N*-methyl acridine orange difluoroheptyl azide (MAO-N_3_, **1**) and *N*-hexyl acridine orange azide (HAO-N_3_, **2**; Fig. 1c), and use them to assemble HIDE probes selective for the IMM. These HIDE probes are benign to mitochondrial health, even under conditions that amplify mitochondrial toxicity. When paired with the appropriate fluorophore, MAO-N_3_ supports long time-lapse imaging of the IMM using confocal, structured illumination microscopy (SIM), SMLM and STED microscopy. When paired with silicon rhodamine (SiR)-DBCO (**3**; Fig. 1d) and detected using confocal microscopy, MAO-N_3_ visualizes the mitochondria for more than 12.5 h with minimal loss in signal intensity or cell viability, whereas the commercial dye MitoTracker Deep Red loses >50% signal intensity within 2 h, and the labeled cells proliferate slowly. When paired with SiR-DBCO and visualized using SIM, MAO-N_3_ visualizes the IMM 3 times longer than the combination of SiR-HaloTag-ligand (SiR-CA) and Halo-TOMM20 and 16 times longer than the fluorescent protein marker mEmerald–TOMM20. When paired with HMDS_655_-DBCO (**4**; Fig. 1d) and visualized using SMLM, MAO-N_3_ distinguishes discrete IMM cristae structures that are not resolved using RhoB-HMSiR, a previously reported HIDE probe assembled using RhoB-TCO and HMSiR-Tz^18^. When paired with SiR-DBCO and visualized using STED microscopy, MAO-N_3_ again improved the visualization of discrete IMM cristae structures relative to RhoB-TCO/SiR-Tz (RhoB-SiR (**7**))^21^ and supported the acquisition of a 125-frame STED movie showing the dynamics of cristae remodeling.

## Results

### HIDE probe design and synthesis

**Extended Data Fig. 1:**
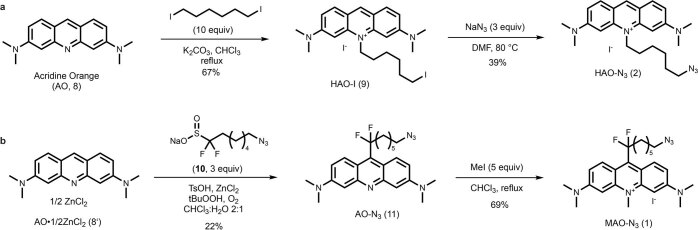
Scheme illustrating the synthetic steps used to prepare HAO-N_3_ and MAO-N_3_. **a**,**b**, Schematic depicting the synthesis of HAO-N_3_ (**a**) and MAO-N_3_ (**b**). Synthetic details are available in Supplementary Information.

The design of IMM-selective HIDE probes was inspired by the properties of nonyl acridine orange (NAO), a small-molecule fluorophore that localizes selectively to the IMM^23^. Selectivity is believed to result from favorable, non-covalent interactions with cardiolipin, an unconventional lipid that is enriched within the IMM^24^. Despite its selective localization, NAO is not photostable and cannot support long time-lapse imaging, especially using modalities that demand high-intensity irradiation^23,25^. We designed two NAO analogs that could assemble HIDE probes suitable for long time-lapse imaging for the IMM: HAO-N_3_ and MAO-N_3_ (Fig. 1c). HAO-N_3_ was synthesized from acridine orange via N-alkylation (Extended Data Fig. 1a)^25^, whereas MAO-N_3_ made use of a Minisci-type radical difluoroalkylation^26^ reaction followed by methylation (Extended Data Fig. 1b).

### In vitro characterization of HAO-N_3_ and MAO-N_3_

**Fig. 2:**
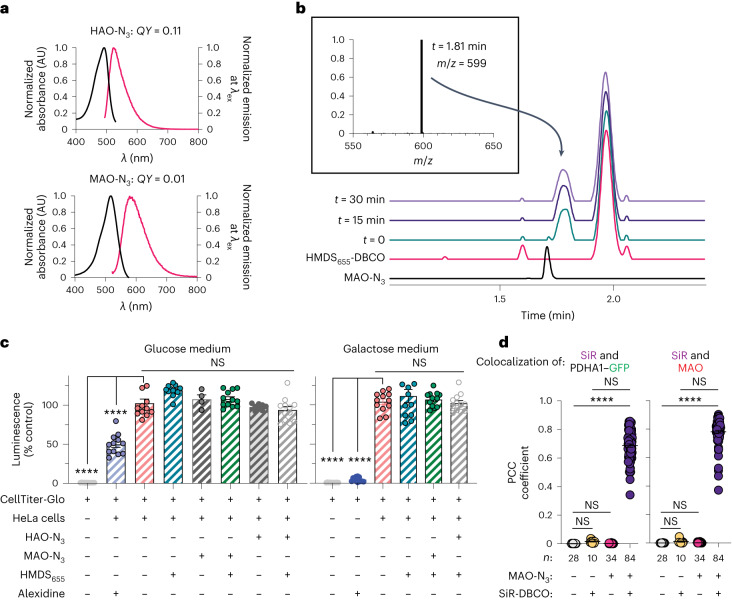
Assembly and characterization of HIDE probes generated using HAO-N_3_ and MAO-N_3_. **a**, Absorption and emission spectra and quantum yield of HAO-N_3_ and MAO-N_3_ at a concentration of 2 µM in Dulbecco’s phosphate-buffered saline (DPBS; pH 7.4, room temperature); AU, arbitrary units. **b**, In vitro SPAAC reaction of 100 µM MAO-N_3_ and 400 µM HMDS_655_-DBCO at 37 °C in deionized water. At the times indicated, 1-µl aliquots of the reaction mixture were withdrawn and analyzed by LC–MS (Methods). The inset shows the mass spectrum confirming the formation of MAO-HMDS_655_ (molecular weight = 1,197.6, *m*/*z* = 599), which elutes at 1.81 min. **c**, HeLa cells incubated in standard glucose-rich medium (DMEM with 4.5 g liter^–1^ glucose supplemented with 10% FBS) or galactose-supplemented oxidative medium (DMEM with 4.5 g liter^–1^ galactose supplemented with 10% FBS) were treated with MAO-N_3_, HAO-N_3_ and/or HMDS_655_-DBCO, and ATP levels were immediately measured (Methods). Plots show the relative bioluminescence signals (percent relative to untreated cells). Untreated cells serve as a negative control, and cells treated with 5 µM alexidine serve as a positive control. Each set of conditions includes 12 biological replicates; ****P < 0.0001; not significant (NS), 0.1137 < *P* < 0.9654 for experiments performed in glucose medium and 0.5886 < *P* < 0.9979 for experiments performed in galactose-supplemented medium. **d**, PCC represents the colocalization of 750 nM SiR-DBCO with 100 nM MAO-N_3_ or PDHA1–GFP (a bona fide mitochondrial matrix marker); PCC_SiR/PDHA1–GFP_ = 0.72 ± 0.01 and PCC_SiR/MAO_ = 0.65 ± 0.01; *n* = 28, 10, 34 or 84 cells for the conditions indicated in the graph sourced from at least two biological replicates. Error bars indicate s.e.m.; ****P < 0.0001; NS, 0.9122 < *P* < 1. Data were analyzed by one-way analysis of variance (ANOVA) with a Dunnett’s post hoc analysis to account for comparisons to the negative control (**c**) or with a Tukey’s multiple comparison test (**d**). Source data

**Extended Data Fig. 2:**
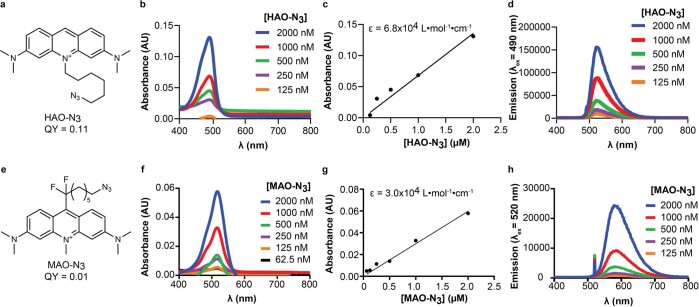
Photophysical properties of MAO-N_3_ and HAO-N_3_. **a**, Structure and quantum yield of HAO-N_3_. **b**, Concentration-dependent UV-Vis absorption curve of HAO-N_3_ shows a λ_max_ of 496 nm. **c**, Plot of absorbance @ λ_max_ against concentration of HAO-N_3_ and the calculated extinction coefficient. **d**, Concentration dependent fluorescence emission of HAO-N_3_ reveals a λ_em_ of 517 nm. **e**, Structure and quantum yield of MAO-N_3_. **f**, Concentration-dependent UV-Vis absorption curve of MAO-N_3_ shows a λ_max_ of 520 nm. **g**, Plot of absorbance @ λ_max_ against concentration of MAO-N_3_ and its extinction coefficient. **h**, Concentration dependent fluorescence emission of MAO-N_3_ reveals a λ_em_ of 570 nm. Source data

We first evaluated the in vitro photophysical properties of HAO-N_3_ and MAO-N_3_ compared to the parent fluorophore NAO. NAO exhibits an absorption maximum at 496 nm, an emission maximum at 519 nm and a quantum yield (*QY*) of 0.16 (ref. ^27^). The photophysical properties of HAO-N_3_ are similar, with an absorption maximum at 496 nm, an emission maximum at 517 nm and a quantum yield of 0.11 (Fig. 2a and Extended Data Fig. 2a–d). By contrast, the absorption and emission curves for MAO-N_3_ were red shifted^28^, with an absorption maximum at 520 nm and an emission maximum at 570 nm (Fig. 2a and Extended Data Fig. 2e–h). MAO-N_3_ (*QY* = 0.01, *ε* = 3.0 × 10^4^ liter mol^−1^ cm^−1^) is also dimmer than HAO-N_3_ (*QY* = 0.11, *ε* = 6.8 × 10^4^ liter mol^−1^ cm^−1^). Both the red shift and lower brightness of MAO-N_3_ are favorable properties for the lipid-like component of a HIDE probe. These properties minimize cross-talk with green fluorescent protein (GFP; *λ*_ex_ = 490 nm and *λ*_em_ = 520 nm) and red fluorescent protein (RFP; *λ*_ex_ = 555 nm and *λ*_em_ = 583 nm) organelle markers and common small-molecule fluorophores that would otherwise complicate multicolor imaging experiments. In vitro experiments verified that an aqueous solution of MAO-N_3_ and HMDS_655_-DBCO reacted within minutes in vitro at 37 °C to generate the expected strain-promoted azide–alkyne click chemistry (SPAAC) product MAO-HMDS_655_ (**5**; Fig. 2b and Supplementary Fig. 1a).

### HIDE probes generated using HAO-N_3_ and MAO-N_3_ are non-toxic

**Extended Data Fig. 3:**
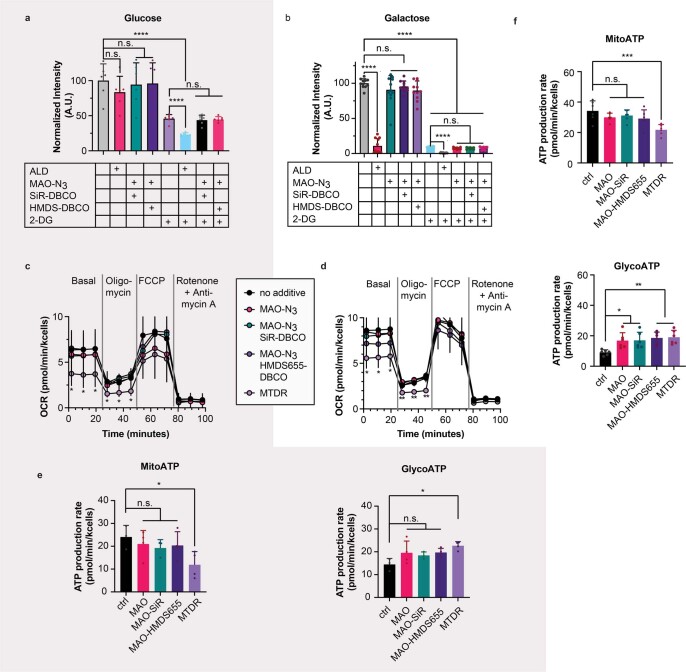
MAO-fluorophore conjugates are benign with respect to multiple metrics of mitochondrial function. **a**,**b**, Plots showing the cellular ATP levels of HeLa cells cultured in glucose-rich (**a**) or galactose-supplemented (**b**) media in the presence or absence of the mitochondrial toxin alexidine (ALD) or the glycolysis inhibitor 2-deoxy-D-glucose (2-DG). HeLa cells were cultured and treated with 2-DG, MAO-N_3_, SiR-DBCO and/or HMDS655-DBCO following protocols in Methods. ATP levels were measured using CellTiter Glo 2.0 bioluminescent assay. *n* = 6 (for **a**) or 12 (for **b**) biologically independent replicates. **** p < 0.0001, n.s. (not significant) 0.5317<p < 0.9994. **c**,**d**, Normalized oxygen consumption rate (OCR) of HeLa cells incubated with MAO-HMDS655, MAO-SiR, MAO-N_3_ or MitoTracker Deep Red (MTDR) in glucose-rich (**c**) or galactose-supplemented (**d**) media under basal conditions and after addition of oligomycin (1 μM) which inhibits ATP synthase, carbonyl cyanide 4-(trifluoromethoxy) phenylhydrazone (FCCP, 10 μM), which disrupts mitochondrial membrane potential, and the combination of rotenone and Antimycin A (Rot + AA, 0.1 μM), which inhibits oxidative phosphorylation. e,f, ATP production rates of cells incubated in glucose-rich (**e**) and galactose-supplemented (**f**) media were calculated based on the measured OCR and extracellular acidification rate (ECAR) using Seahorse Analytics software. *n* = 4 (for **c**–**e**) or 6 (for **f**) biologically independent replicates. For **e**, * *P* = 0.0114 or 0.0391, n.s. (not significant) 0.1184<*P* < 0.8650; for **f**, *** *P* = 0.0004, ** *P* = 0.0031 or 0.0020, * *P* = 0.0170 or 0.0145, n.s. 0.2182<*P* < 0.6294. Error bars = s.e.m. Statistics generated using one-way ANOVA with Dunnett’s post-analysis accounting comparison to the negative control where cells were incubated with cell culture media only. Source data

Next, we evaluated the effect of HAO-N_3_, MAO-N_3_ and HIDE probes derived thereof on the viability of HeLa cells under standard glucose-rich conditions (DMEM with 4.5 g liter^–1^ glucose supplemented with 10% fetal bovine serum (FBS)) and oxidative conditions supplemented with galactose (DMEM with 4.5 g liter^–1^ galactose supplemented with 10% FBS; Fig. 2c and Supplementary Fig. 2). Cells grown in galactose-supplemented medium are reliant on oxidative phosphorylation and are thus sensitive to mitochondrial toxins^29^. Cell viability was evaluated using a commercial assay that detects ATP (CellTiter-Glo 2.0). As expected, cells treated with the known mitochondrial toxin alexidine^30^ displayed diminished ATP levels, and the effects were larger when cells were incubated in oxidative medium containing galactose in place of glucose. The ATP levels of cells treated for 1 h with 100 nM MAO-N_3_ or HAO-N_3_, with or without co-incubation with 750 nM HMDS_655_-DBCO (Fig. 2c) or 750 nM SiR-DBCO (Supplementary Fig. 2), did not differ significantly from the ATP levels of untreated cells. For HeLa cells treated with 2-deoxy-d-glucose, a common glycolysis inhibitor, although a global decrease in ATP levels was observed, no significant changes were present in cells labeled with MAO-N_3_, MAO-HMDS_655_ or MAO-SiR (**6**) compared to in untreated control cells (Extended Data Fig. 3a,b). We also treated cardiomyocytes differentiated from human induced pluripotent stem cells (hiPSC-CMs) with 100 nM MAO-N_3_ followed by 750 nM SiR-DBCO. No apparent effect on the rate of cell body contractions was observed over the course of 110 s (Supplementary Video 1).

To evaluate more quantitatively whether HIDE probes prepared using MAO-N_3_ affect mitochondrial function, we performed real-time respirometry assays (Extended Data Fig. 3c–f). HeLa cells were labeled with MAO-N_3_, MAO-SiR or MAO-HMDS_655_, and oxygen consumption and extracellular acidification rates were measured under multiple energetic states and in both glucose-rich and galactose-supplemented medium. MitoTracker Deep Red, a commercial, carbocyanine-based mitochondria dye with an emission profile similar to SiR (*λ*_ex_ = 644 nm, *λ*_em_ = 665 nm) was also evaluated for comparison purposes. We measured the oxygen consumption rate (OCR) of each labeled cell population under basal conditions as well as under conditions where ATP synthesis was inhibited (using oligomycin), ATP synthesis was maximized (using FCCP) and oxidative phosphorylation was inhibited (using rotenone plus antimycin A; Extended Data Fig. 3c,d).

As anticipated, the measured basal OCR for unlabeled HeLa cells in glucose-rich medium (6.41 ± 0.07 pmol per min per 1,000 cells) was slightly lower than the values measured in galactose-supplemented medium (8.67 ± 0.08 pmol per min per 1,000 cells); both values compare favorably with those from previous reports^29^. Regardless of medium, OCR decreased when ATP synthesis was inhibited with oligomycin, was maximized when the IMM was depolarized by FCCP and decreased to virtually zero when oxidative phosphorylation was inhibited using rotenone plus antimycin A. In both glucose and galactose-supplemented medium, the OCR was diminished by the presence of MitoTracker Deep Red but not by the presence of MAO-SiR or MAO-HMDS_655_. The apparent decrease in maximal OCR in glucose-rich medium after FCCP treatment when cells were labeled with only MAO-N_3_ was not statistically significant and regardless was not observed when the N_3_ functional group was lost after bioconjugation. These studies provide confidence that MAO-SiR and MAO-HMDS_655_ have no measurable effect on mitochondrial function in both standard (glucose-rich) or oxidative (galactose-supplemented) medium.

To fully understand how MAO-generated HIDE probes affect the cellular bioenergy generation dynamics, we also calculated the rate of ATP generation via mitochondrial-related oxidative phosphorylation (mitoATP) and the rate generated by glycolysis (glycoATP) using OCR and extracellular acidification rate values^31^. When using standard glucose-rich medium, no significant changes in OCR, mitoATP or glycoATP were observed regardless of whether cells were treated with MAO-N_3_, MAO-SiR or MAO-HMDS_655_. In comparison, MitoTracker Deep Red-labeled cells exhibited a 57% increase in glycoATP and a 51% decrease in mitoATP, which indicated that this commonly used fluorophore is not benign to oxidative phosphorylation (Extended Data Fig. 3e)^32^. When cells were incubated in galactose-supplemented medium, although all labeled cells showed an increase in glycoATP, only MitoTracker Deep Red-labeled cells showed a 36% decrease in mitoATP (Extended Data Fig. 3f). These data further support the conclusion that MAO-N_3_ or MAO-derived HIDE probes are benign to mitochondrial respiration and ATP generation.

### HIDE probes derived from MAO-N_3_ and HAO-N_3_ localize to mitochondria

**Extended Data Fig. 4:**
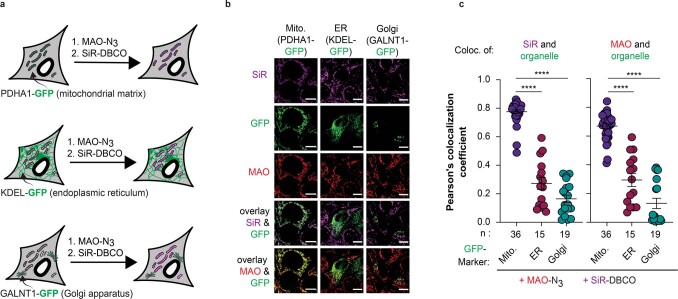
MAO-SiR, the HIDE probe generated from MAO-N_3_ and SiR-DBCO, does not localize significantly to the ER or Golgi. **a**, HeLa cells expressing the mitochondrial matrix marker PDHA1-GFP (CellLight™ Mitochondria-GFP, BacMam 2.0), the endoplasmic reticulum (ER) marker KDEL-GFP (CellLight™ ER-GFP, BacMam 2.0), or the Golgi marker GALNT1-GFP (CellLight™ Golgi-GFP, BacMam 2.0) were treated as described in Methods and imaged on a point-scanning confocal microscope. **b**,**c**, Representative images (**b**) and Pearson’s colocalization coefficients (PCC; **c**) quantifying the colocalization between signals from MAO and SiR with PDHA1-GFP, KDEL-GFP, and GALNT1-GFP, respectively. PCC (MAO/PDHA1-GFP) = 0.66 ± 0.01, PCC (SiR/PDHA1-GFP) = 0.66 ± 0.01; PCC (MAO/GALNT1-GFP) = 0.16 ± 0.03; PCC (SiR/GALNT1-GFP) = 0.13 ± 0.03; PCC (MAO/KDEL-GFP) = 0.27 ± 0.04; PCC (SiR/KDEL-GFP) = 0.29 ± 0.04. Scale bar: 10 μm. *n* = 36, 15, or 19 cells for each condition indicated in the graph, sourced from at least two biological replicates. Error bars = s.e.m. ****P < 0.0001, from one-way ANOVA with Tukey’s multiple comparison test. Source data

**Extended Data Fig. 5:**
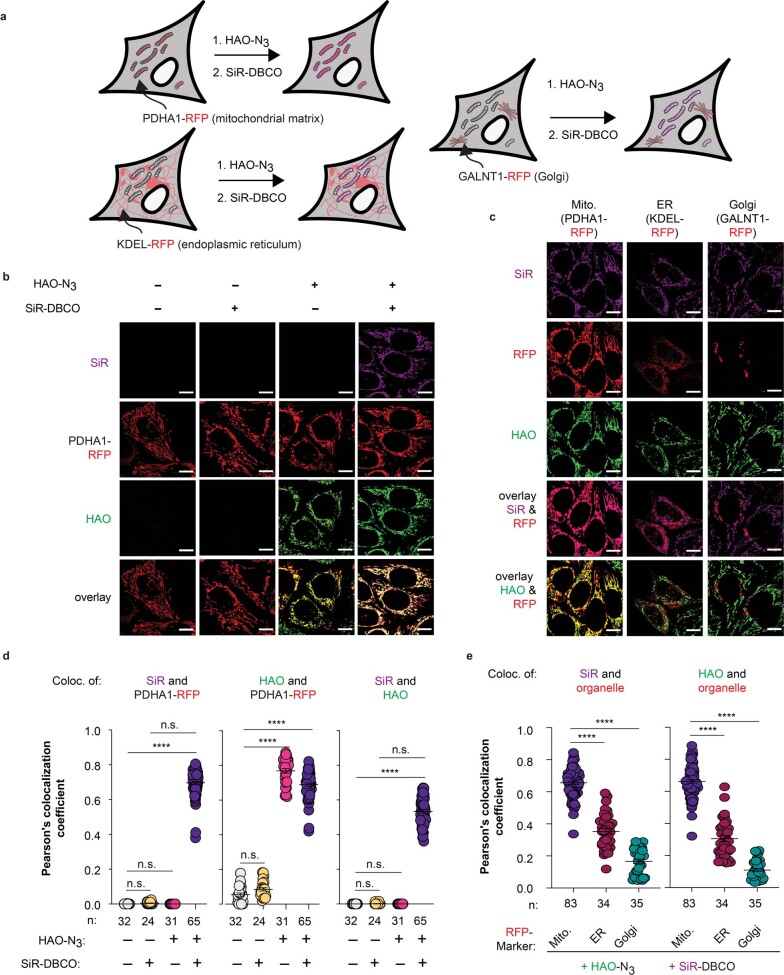
HAO-SiR, the HIDE probe generated from HAO-N_3_ and SiR-DBCO, localizes to mitochondria. **a**, HeLa cells expressing the mitochondrial matrix marker PDHA1-RFP (CellLight™ Mitochondria-RFP, BacMam 2.0), the endoplasmic reticulum (ER) marker KDEL-RFP (CellLight™ ER-RFP, BacMam 2.0), or the Golgi marker GALNT1-RFP (CellLight™ Golgi-RFP, BacMam 2.0) were treated as described in Methods and imaged on a point-scanning confocal microscope. **b**,**c**, Confocal images representing the colocalization signal from HAO-N_3_ or SiR-DBCO with mitochondria marker PDHA1-RFP (**b**) or ER marker KDEL-RFP and Golgi marker GALNT1-RFP (**c**) in HeLa cells. **d**,**e**, Pearson’s colocalization coefficients (PCC) quantifying the colocalization of signal between HAO or SiR and mitochondria, ER or Golgi markers in HeLa cells. PCC(SiR/PDHA1-RFP) = 0.70 ± 0.01, PCC (HAO/PDHA1-RFP) = 0.69 ± 0.01, PCC (SiR/HAO) = 0.53 ± 0.01; PCC (HAO/GALNT1-RFP) = 0.11 ± 0.01; PCC (SiR/GALNT1-RFP) = 0.17 ± 0.01; PCC(HAO/KDEL-RFP) = 0.31 ± 0.02; PCC(SiR/KDEL-RFP) = 0.35 ± 0.02. Scale bar: 10 μm. n = 32, 34, 31, 65, 83, 34, or 35 cells for each condition indicated in the graph, sourced from at least two biological replicates. Error bars = s.e.m. **** *P* < 0.0001, n.s. (not significant) 0.3428<*P* < 0.9999, from one-way ANOVA with Tukey’s multiple comparison test. Source data

Confocal microscopy was used to quantify the extent to which MAO-SiR, assembled in cellula using MAO-N_3_ and SiR-DBCO (Supplementary Fig. 1b), colocalized with a bona fide mitochondrial marker in live HeLa cells. As a marker, we used a fusion of GFP with the leader sequence of pyruvate dehydrogenase-α subunit 1 (PDHA1–GFP), which localizes to the mitochondrial matrix^33^ and whose emission is separate from that of MAO and SiR. Mitochondria in HeLa cells expressing PDHA1–GFP appeared as multiple discontinuous tubular structures emblematic of healthy mitochondria^2,34^ in the presence or absence of 100 nM MAO-N_3_ and/or 750 nM SiR-DBCO (Supplementary Fig. 3b). Cells treated with MAO-N_3_ alone showed notable signal at 570 nm, near the MAO emission maximum, which colocalized with the emission associated with PDHA1–GFP (Pearson’s colocalization coefficient (PCC) = 0.77 ± 0.01; Supplementary Fig. 3c). Cells treated with SiR-DBCO in the absence of MAO-N_3_ showed negligible signal at 660 nm, near the SiR emission maximum. However, cells treated with both MAO-N_3_ and SiR-DBCO showed evidence of signal from SiR, which colocalized well with that of PDHA1–GFP and MAO (PCC = 0.72 ± 0.01 and 0.65 ± 0.01, respectively; Fig. 2d). Although HeLa cells treated with 100 nM NAO and 750 nM SiR-DBCO showed notable signal at 520 nm, near the NAO emission maximum, negligible signal in the SiR channel was observed, illustrating that the azido group of MAO-N_3_ and a subsequent SPAAC reaction^35,36^ is essential to recruit SiR-DBCO to the mitochondria (Supplementary Fig. 4). Additional experiments demonstrated minimal colocalization of MAO-SiR with either the ER or the Golgi (Extended Data Fig. 4) using corresponding bona fide markers (GALNT1–GFP^37^ for the Golgi and KDEL–GFP^38^ for the ER). Analogous experiments using HAO-N_3_, SiR-DBCO and RFP-tagged organelle markers (Extended Data Fig. 5) confirmed that HAO-N_3_ is also capable of mitochondria-specific labeling. We conclude that HAO-N_3_ and MAO-N_3_ localize selectively to mitochondria in live HeLa cells, and formation of the corresponding HIDE probe after SPAAC reaction with SiR-DBCO localizes SiR to the mitochondria.

**Fig. 3:**
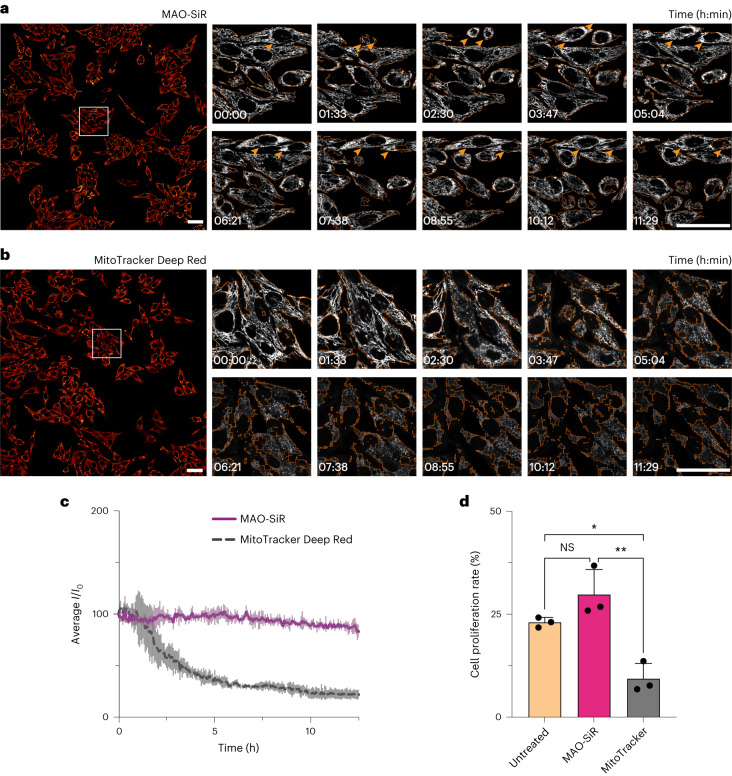
Long time-lapse confocal imaging using MAO-SiR or MitoTracker Deep Red. **a**,**b**, HeLa cells were labeled with 200 nM MAO-N_3_ and 100 nM SiR-DBCO (**a**) or 100 nM MitoTracker Deep Red (**b**) and imaged using a point-scanning confocal microscope. In total, 220 frames of 670 × 670 µm^2^ images were obtained over 12.5 h. Individual frames from regions of interest (ROIs; white rectangles) are shown to the right. Orange outlines indicate segmentation boundaries used to isolate signals for intensity quantification. Orange arrowheads in **a** represent observable cell division events; scale bar, 50 µm. **c**, Plots of average signal decay over time. Mean signals from segmented regions were averaged to determine the mean frame intensity. Mean frame intensities from each dataset were averaged and plotted for each time point along with the relative standard deviation; *n* = 3 biologically independent replicates; *t*_1/2_ (MitoTracker) = 1.96 ± 0.05 h; *t*_1/2_ (MAO-SiR) = not applicable. Intensities (*I*) for plots in **c** were scaled relative to the first frame’s intensity (*I*_0_) in the data sets. **d**, Cell proliferation rates for untreated HeLa cells or HeLa cells labeled with MAO-SiR or MitoTracker Deep Red over the entire field of view over 12.5 h; *n* = 3 biologically independent replicates. Error bars represent s.e.m.; **P = 0.0017; *P = 0.0122; NS, *P* = 0.1542. Data in **d** were analyzed by one-way ANOVA with Tukey’s multiple comparison test. Source data

### MAO-SiR supports overnight confocal imaging of mitochondria

We labeled HeLa cells with 200 nM MAO-N_3_ and 100 nM SiR-DBCO and obtained a 220-frame confocal time-lapse series with a field of view of 670 × 670 μm^2^ over the course of 12.5 h (Fig. 3a and Supplementary Video 2). Each frame in the time series was reconstructed by stitching 16 individual frames together. Over this time period, HeLa cells labeled with MAO-SiR exhibited negligible signal decay and increased in density by 27 ± 6%, which compares well with an increase of 23 ± 1% observed for untreated cells (Fig. 3c,d and Supplementary Video 3)^39^. By contrast, cells labeled with 100 nM MitoTracker Deep Red, a commercial carbocyanine-based mitochondria dye with a similar emission profile (*λ*_ex_ = 644 nm, *λ*_em_ = 665 nm), increased in density by only 9 ± 4%, and the signal intensity decreased by 50% within 2 h (Fig. 3b–d and Supplementary Video 4). Although previous work demonstrated that HIDE probes increase the length of time over which bright images can be acquired using SMLM and STED microscopy^15,18–22^, these experiments reveal that comparable improvements are seen even during microscopy using lower-intensity irradiation.

### MAO-SiR labels the inner mitochondrial membrane even after membrane depolarization

**Extended Data Fig. 6:**
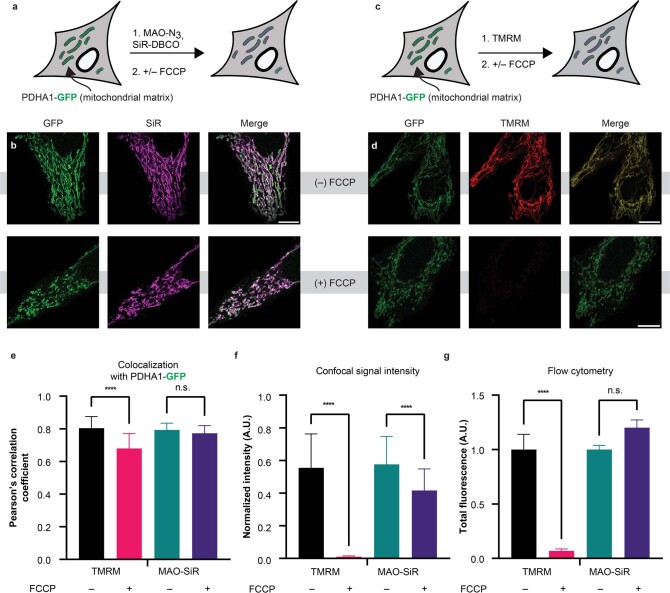
MAO-SiR visualizes mitochondria under depolarizing conditions induced by FCCP. **a**, HeLa cells expressing PDHA1-GFP were labeled with MAO-SiR (200 nM MAO-N_3_ + 100 nM SiR-DBCO), treated with or without FCCP (10 µM, 30 min), and imaged using confocal microscopy; **b**, Representative confocal images of MAO-SiR-labeled cells with or without FCCP treatment. **c**, HeLa cells expressing PDHA1-GFP were labeled with tetramethylrhodamine methyl ester (TMRM, 100 nM), treated with or without 10 µM FCCP, and imaged using confocal microscopy. **d**, Representative confocal images of TMRM-labeled HeLa cells with or without FCCP treatment. **e**–**g**, Effect of FCCP on the colocalization of PDHA1-GFP with TMRM or SiR (**e**), normalized signal intensity from confocal images (**f**) or total fluorescence as determined by flow cytometry (**g**). *n* = 3 biologically independent replicates for experiments shown in **e**–**g**. Scale bar: 10 µm. Error bars = s.e.m. ****  *P* < 0.0001, n.s. (not significant) *P* = 0.3590 (for **e**) or 0.0634 (for **g**), from one-way ANOVA with Šídák’s multiple comparisons test. Source data

Many IMM and mitochondria-targeting probes, such as PKMO^14^, tetramethylrhodamine methyl ester (TMRM)^23^ and RhoB-SiR (**7**)^21^, label mitochondria on the basis of membrane potential. As a result, none of these probes are suitable for imaging depolarized mitochondria. Because MAO-SiR is believed to label mitochondria via an interaction with specific lipids (a depolarization-resistant mechanism), we hypothesized that it would be better retained within depolarized mitochondria and not be lost, as is observed for probes retained on the basis of membrane potential. To test this hypothesis, we labeled HeLa cells with MAO-SiR and depolarized the cells with FCCP, a well-known mitochondrial membrane uncoupler that rapidly shuttles protons across the IMM (Extended Data Fig. 6a,b)^40^. TMRM, which is retained in mitochondria on the basis of membrane potential^41^, was used as a control (Extended Data Fig. 6c,d). The fluorescent signal from MAO-SiR was retained within mitochondria for at least 30 min after FCCP treatment (Extended Data Fig. 6b,e–g). By contrast, the fluorescent signal due to TMRM dropped by 98% within 30 min after depolarization with FCCP (Extended Data Fig. 6d,f,g).

### Structured illumination microscopy imaging of the mitochondria using SiR-DBCO and MAO-N_3_

**Fig. 4:**
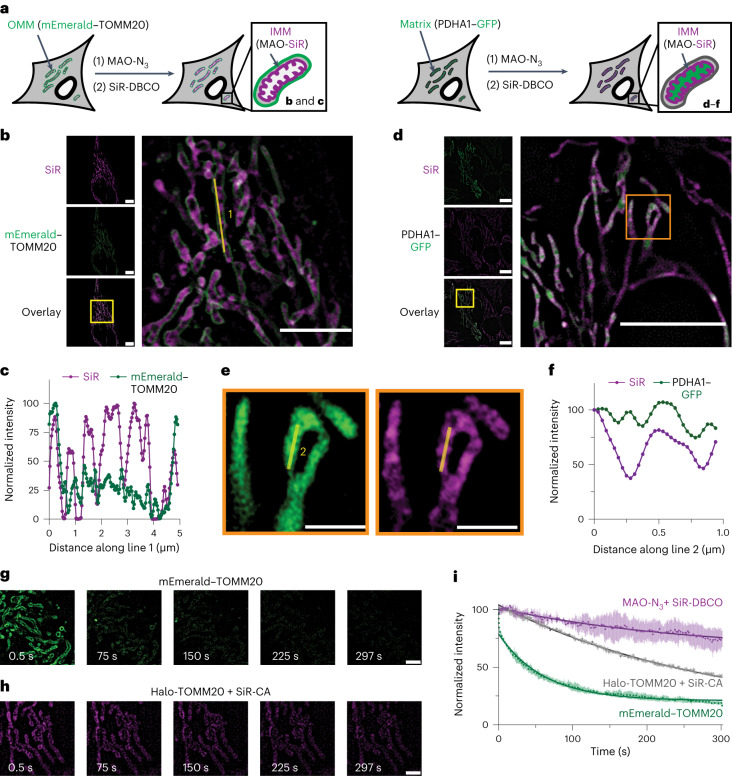
SIM imaging of mitochondria using MAO-SiR. **a**, HeLa cells expressing PDHA1–GFP or mEmerald–TOMM20 were treated with 75 nM MAO-N_3_ and 600 nM SiR-DBCO (Methods) and imaged using lattice SIM. **b**, Representative SIM images of cells expressing mEmerald–TOMM20 and labeled with MAO-SiR as described in **a**; scale bar, 5 µm. **c**, Plots of signal intensity from SiR and mEmerald along line 1. **d**, Representative SIM images of cells expressing PDHA1–GFP and labeled with MAO-SiR as described in **a**; scale bar, 5 µm. **e**, Enlarged image of the region indicated by the orange box in **d**. **f**, Plots of signals from SiR and GFP along line 2; *n* = 3 biologically independent replicates for the data shown in **b**–**f**. **g**,**h**, Snapshots of selected ROIs from Supplementary Videos 6 and 7 obtained from HeLa cells expressing mEmerald–TOMM20 (**g**; Supplementary Movie 6) or SiR-TOMM20 (**h**; Supplementary Movie 7) at the indicated times; scale bar, 2 μm. **i**, Plots illustrating normalized SiR fluorescence signals of SiR-MAO and SiR-TOMM20 and GFP signal from mEmerald–TOMM20 over time. Error bars represent s.e.m.; *n* = 3 ROIs; *N* = 1 cell. Intensities for plot **i** were scaled relative to the first frame’s intensity in the data sets. Source data

Having confirmed by confocal microscopy that the HIDE probe MAO-SiR is non-toxic and mitochondria specific and supports extended-time imaging, we turned to higher-resolution methods to evaluate its intraorganelle localization. HeLa cells expressing either mEmerald–TOMM20 (an OMM marker) or PDHA1–GFP (a mitochondrial matrix marker) were incubated with 75 nM MAO-N_3_ and 600 nM SiR-DBCO and imaged using lattice SIM (Fig. 4a). Images of treated cells expressing mEmerald–TOMM20 show clear differentiation of the OMM (mEmerald) and internal mitochondrial fine structure (SiR; Fig. 4b,c). Images of treated cells expressing PDHA1–GFP (mitochondrial matrix marker) also show differences in signal localization (Fig. 4d–f). Although the profile of the signals due to PDHA1–GFP are relatively continuous, those due to MAO-SiR are not (Fig. 4e,f), revealing a discontinuous structure that resembles those seen in Fig. 4b. Due to the limited resolution of SIM (Supplementary Fig. 5a–d), the signals from the SiR channel (full-width at half-maximum (FWHM) = 171 ± 9 nm; Supplementary Fig. 5d) cannot fully resolve the cristae topology of the IMM^13,42^. Despite this limitation, these SIM experiments provide strong evidence that the HIDE probe MAO-SiR labels neither the OMM nor the mitochondrial matrix.

**Extended Data Fig. 7:**
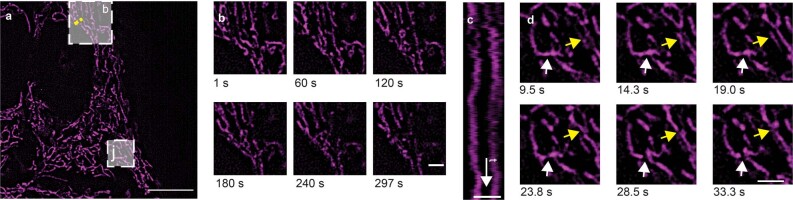
Long time-lapse SIM imaging of IMM dynamics using the HIDE probe MAO-SiR in HeLa cells. **a**–**d**, Representative images from a 500-frame SIM series obtained over 297 s (Supplementary Movie 5). **a**, SIM image at t = 0.5 s. Scale bar: 10 μm. **b**, Snapshots of the bracketed region indicated in a at the times indicated. Scale bar: 2 μm. **c**, Kymograph of the yellow dashed line in a. Scale bar: 1 μm. **d**, IMM fission (white arrow) and fusion (yellow arrow) events from t = 9.5 s to t = 33.3 s in the bracketed region in **a**. Scale bar: 2 μm.

**Extended Data Fig. 8:**
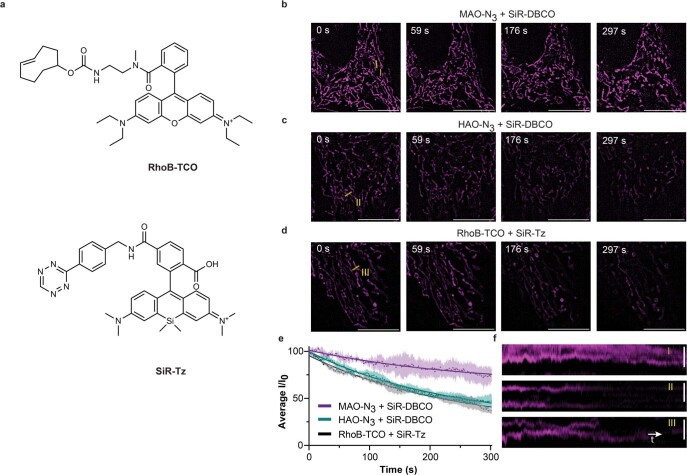
The IMM-selective HIDE probe supports extended-time SIM imaging in live cells. **a**, Structures of RhoB-TCO and SiR-Tz. **b**–**d**, HeLa cells were treated with MAO-N_3_ (**b**), HAO-N_3_ (**c**) or RhoB-TCO (**d**) probes followed by SiR-DBCO (**b** and **c**) or SiR-Tz (**d**) as described in Methods, and imaged on a widefield microscope equipped with lattice SIM. Scale bar: 10 μm. **e**, Plot illustrating normalized SiR fluorescence signals of MAO-SiR, HAO-SiR and RhoB-SiR over time. t_1/2_ (HAO-SiR) = 248.2 ± 1.2 s; t_1/2_ (RhoB-SiR) = 233.0 ± 1.3 s. *n* = 3 regions of interest (ROI), *N* = 1 cell. Error bar = s.e.m. **f**, kymograph of line I (MAO-SiR), II (HAO-SiR), and III (RhoB-SiR). Scale bar: 1 μm. Source data

To evaluate the time over which interpretable images can be acquired using SIM, we treated HeLa cells with MAO-N_3_ and SiR-DBCO as described above and imaged them for 297 s (500 frames; Extended Data Fig. 7a–d and Supplementary Video 5). Over a field of view sufficient to visualize an entire cell (Extended Data Fig. 7a), this lattice SIM movie captured both IMM fission (white arrow) and fusion (yellow arrow) events (Extended Data Fig. 7d) with no change in mitochondrial morphology (Extended Data Fig. 7b) and excellent signal retention (Extended Data Fig. 7c). When cells were imaged using mEmerald–TOMM20, little or no signal was observed after 75 s (Fig. 4g and Supplementary Video 6). When cells expressing Halo-TOMM20-N-10 were labeled with SiR-CA to generate the fluorophore–protein conjugate SiR-TOMM20, significant signal loss was also evident (Fig. 4h and Supplementary Video 7). Exponential decay analyses of intensity data revealed a signal half-life of 713.5 ± 8.0 s for MAO-SiR, which is 3.2 times as long as that of SiR-TOMM20 (*t*_1/2_ = 224.4 ± 0.6 s) and 16 times longer than mEmerald–TOMM20 (*t*_1/2_ = 42.4 ± 0.5 s; Fig. 4i). The signal half-lives of HAO-SiR (*t*_1/2_ = 248.2 ± 1.2 s) and the previously reported mitochondrial HIDE probe RhoB-SiR (*t*_1/2_ = 233.0 ± 1.3 s) were both also significantly smaller than MAO-SiR (Extended Data Fig. 8).

### Single-molecule localization microscopy imaging of the mitochondria using HMDS_655_-DBCO and MAO-N_3_

**Fig. 5:**
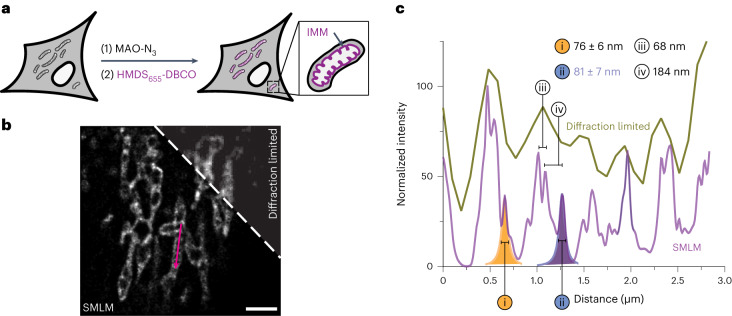
SMLM imaging of the IMM using a HIDE probe assembled from MAO-N_3_ and HMDS_655_-DBCO (MAO-HMDS_655_). **a**, HeLa cells were treated with 75 nM MAO-N_3_ and 600 nM HMDS_655_-DBCO and imaged using a widefield microscope with SMLM capabilities. **b**, Representative SMLM image reconstructed with 800 frames (*t* = 4 s). The corresponding diffraction-limited image is shown for comparison; scale bar, 2 μm; *n* = 3 biologically independent replicates. **c**, Plot of signal intensity along the magenta arrow in **b** in both the reconstructed SMLM and widefield images. Two peaks in the SMLM image, labeled i and ii, were fitted into a Lorentzian function to obtain FWHM values of 76 ± 6 nm and 81 ± 7 nm, respectively. Distances (iii and iv) between two pairs of adjacent peaks were measured from centroid to centroid, giving values of 68 nm and 184 nm, respectively. Source data

We next used an MAO-derived HIDE probe and SMLM to visualize the fine structure of the IMM. HeLa cells were treated with 75 nM MAO-N_3_ for 40 min, washed, incubated for 20 min with 600 nM HMDS_655_-DBCO^43^ (Fig. 5a) and imaged using SMLM in an antioxidant-supplemented buffer^18^. A representative image constructed from 800 frames (*t* = 4 s) is shown in Fig. 5b. The low temporal resolution made it challenging to capture the fine structure of continuously moving mitochondria^44^. Nevertheless, the images reveal multiple tubular cristae structures in a ladder-like pattern; these features were not resolved by a previously reported HIDE probe generated using RhoB-TCO and HMSiR-Tz^18^. Fourier ring correlation analysis was performed, which yielded a global resolution of 161.7 nm (ref. ^45^). The resolution of the images using MAO-HMDS_655_ is sufficiently high (FWHM values of 76 ± 6 nm and 81 ± 7 nm for the representative peaks in the line profile; Fig. 5c) to establish distances between adjacent cristae of 68 nm to 184 nm, comparable to previous reports^13,42^.

### Stimulated emission depletion imaging of inner mitochondrial membrane cristae enabled by SiR-DBCO and MAO-N_3_

Having validated that HIDE probes assembled using MAO-N_3_ offer tangible improvements in the time over which IMM images can be generated using both SIM and SMLM, we turned to the toughest test, STED microscopy. STED microscopy offers greater spatial resolution than SIM and greater temporal resolution than SMLM and is thus well suited to reveal rapid dynamics in diffraction-limited cellular compartments, such as the IMM. Previous IMM markers that support STED imaging, such as SiR-COX8A^46^, MitoPB Yellow^13^, PKMO^14^ and MitoESq-635 (ref. ^9^), are limited by photostability or photocytotoxicity.

**Fig. 6:**
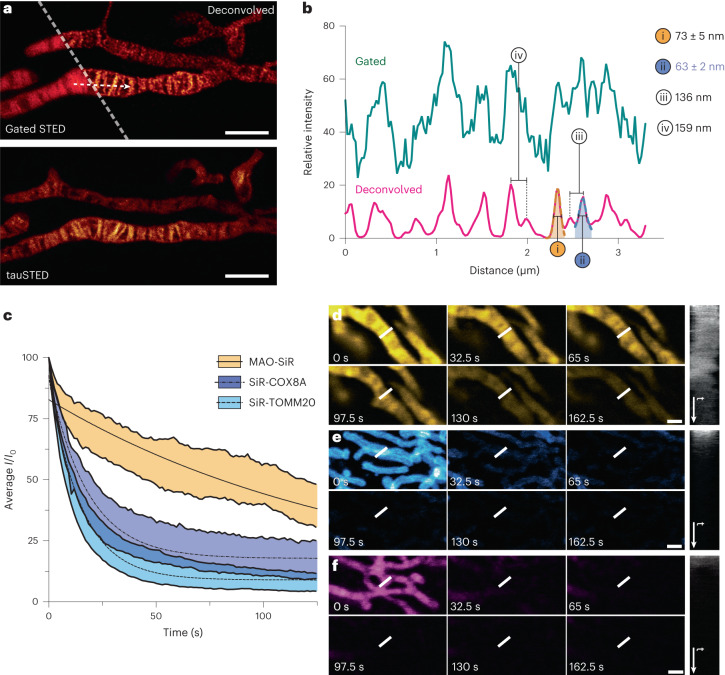
STED imaging of the IMM using MAO-SiR. **a**, Top, gated and deconvolved STED image. Bottom, tauSTED image generated using fluorescence lifetime filtering. Images here are representative of at least three independent replicates; scale bar, 2 μm. **b**, Plot of SiR signal intensity along the white arrow in **a** in the gated STED and deconvolved STED image. Two peaks in the STED image were fitted into a Lorentzian function to obtain FWHM values of 73 ± 5 nm (**i**) and 63 ± 2 nm (**ii**), respectively. Distances between pairs of adjacent peaks were also measured from centroid to centroid, giving values of 136 nm (**iii**) and 159 nm (**iv**), respectively. **c**, Plots illustrating relative SiR fluorescence signals of SiR-MAO, SiR-SNAP/COX8A-SNAP (SiR-COX8A) and SiR-CA/Halo-TOMM20 (SiR-TOMM20) over 125 frames obtained in 162.5 s; *n* = 7 fields of view over *N* = 7 individual cells. Intensities for **c** were scaled relative to the first frame’s intensity in the data sets. Error bars represent s.e.m. **d**–**f**, Selected STED images using MAO-SiR (**d**, yellow; Supplementary Video 8), SiR-TOMM20 (**e**, blue; Supplementary Video 9) or SiR-COX8A (**f**, magenta; Supplementary Video 10), each from a 125-frame, 162.5-s movie at the indicated time points. The white bar in each frame indicates the ROI for the kymograph on the right. Kymographs are plotted to the right of each frame and are set to track the intensity of the signal within the ROI for each frame in the movie; scale bar, 1 μm. Source data

**Extended Data Fig. 9:**
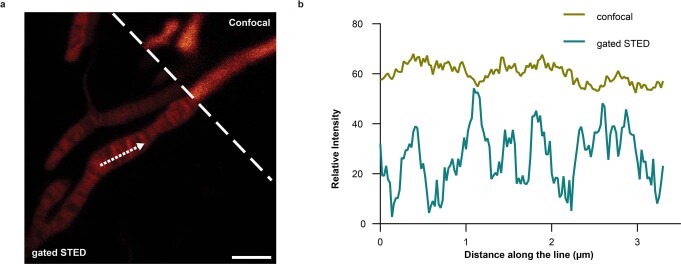
Comparison of diffraction limited confocal microscopy vs. time-gated STED microscopy when imaging the IMM using MAO-SiR. **a**, Gated STED and confocal images of signals due to MAO-SiR, representative from 3 biologically independent replicates. Scale bar: 2 μm. **b**, Plots of signals due to SiR in confocal and STED channels along the dotted arrow in **a**. Source data

We first examined different data acquisition modes in STED microscopy using HeLa cells labeled with MAO-SiR. We acquired images of the cristae using gated STED and deconvolved the images using a theoretical point spread function (FWHM up to 63 ± 2 nm; Fig. 6a, top). We then filtered the fluorescence signals on the basis of fluorescence lifetime in tauSTED mode^47^ and acquired images of the IMM with excellent resolution (Fig. 6a, bottom). These images clearly demark densely packed, ladder-like mitochondrial cristae with regular dimensions (Fig. 6b) that are indiscernible by confocal microscopy (Extended Data Fig. 9a,b). Moreover, the fluorescent signal from MAO-SiR (*t*_1/2_ = 112.7 ± 2.1 s) diminished five times more slowly than signal from SiR-COX8A (*t*_1/2_ = 17.4 ± 0.4 s) and ten times more slowly than SiR-TOMM20 (*t*_1/2_ = 10.4 ± 0.2 s; Fig. 6c). This apparent photostability supported the acquisition of a 162.5-s movie of cristae dynamics containing 125 tauSTED frames (Fig. 6d–f). Distinct cristae are visible in images obtained with MAO-SiR until the last frame without the need for genetic manipulation (Fig. 6d and Supplementary Video 8), whereas images obtained with SiR-COX8A and SiR-TOMM20 are lost within 65 s (Fig. 6e,f and Supplementary Videos 9 and 10).

## Discussion

The mitochondria is a highly dynamic organelle that undergoes coordinated and balanced cycles of fission and fusion to maintain its shape, content, transport ability and size. These transient and rapid morphological changes allow mitochondria to meet metabolic demands, initiate the degradation of damaged organelles and contribute to control of the cell cycle, immunity and cellular apoptosis. Defects in mitochondrial dynamics cause disease^48^. Decoupled fusion and fission and resulting mitochondrial fragmentation occur in response to stress and during heart failure^49^, neurodegenerative disease, cancer and obesity. Recent studies have shown that the dynamics of the IMM are critical, for example, for the loss of mitochondrial DNA from the matrix into the cytosol to trigger the innate immune cGAS–STING pathway^50^.

Imaging the mitochondria to detect dynamic morphological changes (especially those involving the complex inner membrane) is especially challenging, as the subcompartment is replete with enzymes that generate dye-inactivating reactive oxygen species. Moreover, the imaging process itself can damage essential macromolecules. These challenges are exacerbated when one seeks to evaluate changes in mitochondrial dynamics or interactions that occur over notable time frames in primary or human-derived cells and/or using super-resolution imaging modalities that require intense irradiation. One proven strategy to overcome these challenges involves labeling the organelle membrane lipids as opposed to the proteins embedded within^15–22,51^. Labeling the membrane lipids positions the dye at high density within a hydrophobic environment in which most dye molecules exist in a dark state and do not absorb light. Balancing the labeling density with the fraction of molecules in the dark state produces bright images that last because the dark-state molecules act as a reservoir to replenish dyes that bleach.

Here, we describe new HIDE probes that selectively image an organelle subcompartment (the IMM) in live cells and over exceptionally long time frames. These probes, all assembled from the lipid-like molecule MAO-N_3_, are photostable, lack detectable toxicity and support exceptional spatial resolution of the IMM using multiple modalities, all without genetic manipulations. Confocal images of the mitochondria generated using MAO-SiR remained stable for more than 12.5 h with little or no loss in signal intensity or cell viability, more than six times longer than possible using MitoTracker Deep Red. SIM images of the IMM using MAO-SiR lasted for 16 times longer than those obtained using mEmerald–TOMM20 and supported the acquisition of 500 frames over the course of 5 min, as opposed to 20 frames over 2.8 min possible with MitoTracker Green^52^. When paired with HMDS_655_-DBCO (Fig. 1d) and visualized using SMLM, MAO-N_3_ distinguished discrete IMM cristae structures that were not resolved using a previously reported HIDE probe assembled using RhoB-TCO^18^. When paired with SiR-DBCO and visualized using STED microscopy (Fig. 1d), MAO-N_3_ again improved the visualization of discrete IMM cristae structures relative to RhoB-TCO^21^ and supported the acquisition of a 125-frame STED movie showing cristae dynamics. HIDE probes based on MAO-N_3_ are versatile, non-toxic, photostable and completely cell permeant. These features should make them highly useful, alone or in combination with other organelle markers, for long-term analysis of natural and pathological mechanisms related to IMM dynamics and interorganelle interactions.

## Methods

### Materials

SiR-CA was synthesized according to previously reported methods. BacMam 2.0 reagent, CellLight Mitochondria-GFP (PDHA1–GFP; C10600), CellLight Mitochondria-RFP (PDHA1–RFP; C10505), CellLight Golgi-GFP (GALNT1–GFP; C10592), CellLight Golgi-RFP (GALNT1–RFP; C10593), CellLight ER-GFP (KDEL–GFP; C10590), CellLight ER-RFP (KDEL–RFP; C10591), culturing medium DMEM (12430054, 21063029 and A1443001), Opti-MEM with no phenol red (11058021) and FBS and chemical reagents Hoechst 33342 (62249) and MitoTracker Deep Red FM (M22426) were all purchased from Thermo Fisher. CellTiter-Glo 2.0 (G9242) was purchased from Promega, and alexidine dihydrochloride (A8986) was purchased from Sigma-Aldrich. Plasmid encoding mEmerald–TOMM20 was a gift from M. Davidson (Florida State University, mEmerald–TOMM20-N-10, Addgene plasmid 54282). Plasmid encoding Halo-TOMM20 was a gift from K. McGowan (HHMI Janelia, Halo-TOMM20-N-10, Addgene plasmid 123284). Plasmid encoding COX8A-SNAP was a gift from A. Egana (New England Biolabs, pSNAPf-COX8A, Addgene plasmid 101129).

### Cell culture

HeLa cells (University of California, Berkeley, Cell Culture Facility) were cultured in DMEM (Thermo Fisher) supplemented with 10% FBS, penicillin (100 U ml^–1^) and streptomycin (100 μg ml^–1^). All cells were cultured at 37 °C in a humidified CO_2_/air (5%/95%) incubator. All cells were bona fide cell lines obtained from the University of California, Berkeley, Cell Culture Facility and were periodically tested for *Mycoplasma* by using DNA methods. Cells for imaging were seeded in four- or eight-well LabTek chambers (Nunc, Thermo Fisher, 177399 and 177402, number 1.0, 0.8 cm^2^ for four-well chambers and 1.8 cm^2^ for eight-well chambers; these chambers were used for confocal colocalization studies, SIM and SMLM) or four-well glass-bottom μslides (Ibidi, 80427, number 1.5H, 2.5 cm^2^; these μslides were used for STED microscopy and overnight confocal imaging) at the indicated densities.

### Microscopy

Colocalization studies were performed on a Zeiss LSM 880 confocal microscope equipped with a Plan-Apo ×63/1.4-NA (Zeiss) oil objective and a diode 405-nm laser, an argon 458-, 488- and 514-nm laser, a diode pumped solid-state 561-nm laser and a 633-nm HeNe laser with standard settings. The pinhole size was set to 1 Airy unit.

Overnight time-lapse confocal and STED microscopy were performed on a Leica Stellaris 8 microscope (Leica Microsystems) equipped with a Leica DMi8 CS scanhead, an HC Plan-Apo ×100/1.4-NA STED White oil immersion objective (for STED microscopy), an HC Plan-Apo ×63/1.4-NA oil immersion objective (for point-scanning confocal time-lapse imaging), an HC Plan-Apo ×63/1.4-NA water immersion objective (for point-scanning confocal hiPSC-CM imaging), a pulsed white-light laser (440–790 nm; 440 nm: >1.1 mW; 488 nm: >1.6 mW; 560 nm: >2.0 mW; 630 nm: >2.6 mW; 790 nm: >3.5 mW, 78 MHz) and a pulsed 775-nm STED laser. Confocal imaging was performed using either HyD S or HyD X detectors in analog or digital mode, whereas STED imaging was exclusively performed on HyD X detectors in photon counting mode. Live-cell imaging conditions were maintained using a blacked out cage enclosure from Okolab. The temperature was maintained by heating the enclosure and was monitored by using Oko-Touch. The pH was maintained by supplying humidified 5% CO_2_ to the sample chamber.

Lattice SIM and SMLM imaging were performed on an inverted Zeiss Elyra 7 microscope equipped with a Plan-Apo ×63/1.46-NA oil immersion objective (Zeiss) and a 403-nm (200-mW) laser, a Sapphire 488-nm (1-W) and 561-nm (1.3-W) laser and a Lasos 642-nm (550-mW) laser, MBS 405/488/561/641 and EF LBF 405/488/561/641 filter sets, LP 560 and BP 570–620 + LP 655 filter cubes and two pco.edge high-speed sCMOS cameras using Zeiss Zen Black software.

### Probe synthesis and characterization

Experimental protocols for the synthesis of MAO-N_3_ and data representing the characterization of MAO-N_3_, HAO-N_3_ and HMDS_655_-DBCO, in vitro spectroscopic characterization, the SPAAC reaction rate study, cell viability and real-time respirometry studies are provided in the Supplementary Notes.

### Confocal imaging to monitor cell body contraction of human induced pluripotent stem cell-derived cardiomyocytes

hiPSC-CMs were prepared according to a previously reported procedure^53^ and were cultured on 24-well μ-plates (Ibidi, 82426) coated with Matrigel (1:100 dilution; Corning; 7.0 × 10^4^ cells per well) in RPMI 1640 medium (Life Technologies) with B27 supplement (Life Technologies). On the day of imaging, the hiPSC-CMs were labeled with 600 μl of 100 nM MAO-N_3_ in RPMI 1640/B27 without phenol red (ph(–)) for 1 h, washed three times with 600 μl of RPMI 1640/B27 ph(–), incubated with 600 μl of 0.75 μM SiR-DBCO in RPMI 1640/B27 ph(–) for 1 h and washed again six times with RPMI 1640/B27 ph(–) for 10 min each. Finally, the cells were imaged in 700 μl of RPMI 1640/B27 ph(–) at 37 °C and 5% CO_2_. In total, 213 frames from a field of view of 72.5 μm × 72.5 μm were obtained with a time interval of 0.52 s and a time lapse of 110 s. The pixel size was 0.142 μm, pixel dwell time was 0.95 μs, scan speed was 1,000 Hz, and laser power was 25.7% (652 nm).

### Confocal imaging for colocalization with organelle markers

Two days before imaging, HeLa cells were seeded in LabTek I eight-well chambers (1.5 × 10^4^ cells per well). After 24 h, 6 µl of a CellLight BacMam reagent (40 particles per cell) for organelles of interest (ER, Golgi or mitochondria) was added to each well, and incubation was continued for 16–20 h. The cells were subsequently treated with 300 μl of 100 nM HAO-N_3_ or MAO-N_3_ (diluted from 0.2 mM DMSO stock solution into DMEM ph(–) buffer) for 1 h. The cells were washed three times with DPBS and treated with 300 μl of a 750 nM solution of SiR-DBCO in DMEM ph(–) for 1 h. Each well was then washed with 300 µl of DMEM supplemented with 10% FBS and incubated for 10 min. The wash/incubation was repeated six times to minimize non-specific dye labeling. Cells were then incubated with 300 µl of 0.5 µg ml^–1^ Hoechst 33342 (Thermo Fisher, 62249) solution for 1 min to label nuclei and help identify the plane of view. Cells were washed with warm DPBS and imaged in DMEM ph(–) at room temperature. Pairwise colocalization of HAO/MAO/SiR/GFP markers was evaluated by measuring the overlap of fluorescent signals from two spectrally separated channels using the PCC for individual cells and the JaCOP plugin in Fiji. PCC values for each condition obtained from multiple cells collected over at least two biological replicates were pooled and are represented as mean and s.e.m. using Prism 8.4.3.

### Overnight confocal imaging for cell proliferation monitoring

Forty-eight hours before imaging, HeLa cells (P3-P10) were seeded in a four-well chamber (2.2 × 10^4^ cells per well). On the day of the experiment, the cells were treated with 700 μl of a 200 nM solution of MAO-N_3_ (diluted from 0.2 mM DMSO stock solution in Opti-MEM ph(–) buffer) for 1 h. The cells were washed one time with DMEM ph(–) and treated with 700 μl of a 100 nM solution of SiR-DBCO (diluted from 2 mM DMSO stock solution into Opti-MEM ph(–) buffer) for 1 h. The cells were then washed with 700 µl of DMEM supplemented with 10% FBS and incubated in the same buffer for 1 h. For the experiment with MitoTracker Deep Red, cells were treated with 700 μl of a 100 nM solution of MitoTracker Deep Red (diluted from 0.2 mM DMSO stock solution into Opti-MEM ph(–) buffer) for 30 min and washed three times with 700 μl of DPBS. For untreated cells, the cells were washed three times with 700 μl of DPBS. Finally, the cells were imaged in 700 μl of DMEM ph(–) with 10% FBS at 37 °C and 5% CO_2_.

A region of 4 × 4 frames was imaged every 2 min, and each time point in the time lapse was derived by stitching the 16 frames together. Each time point captured a field of view of 670 μm × 670 μm. For each frame within the time point composite, the field of view was 184.52 × 184.52 μm. The focal plane was maintained using Leica’s Adaptive Focus Control system. The frame size was 1024 × 1024, the pixel size was 0.18 μm, the pixel dwell time was 2.8375 μs, and the detection window was 662–749 nm. The laser power was 10% (652 nm) for MAO-SiR-treated cells and untreated cells and 2% for MitoTracker Deep Red-treated cells. Cell counts of frame 1 and frame 220 were performed manually using the Cell Counter plugin in Fiji. Segmentation was run to isolate cell bodies from background, and a mask was generated to identify ROIs for intensity quantification. Mean signal for each ROI was averaged for one time point to give the mean frame intensity. Each mean frame intensity was scaled relative to the first frame of the time series. The mean for each frame was then averaged over three data sets to determine the half-life. Half-life of signal intensity was obtained via performing a nonlinear regression of the exponential decay function using OriginLab OriginPro 2022b.

### Confocal imaging and flow cytometry of cells with depolarized mitochondria

Forty-eight hours before imaging, HeLa cells (P3-P10) were seeded in a four-well chamber (2.2 × 10^4^ cells per well). After 24 h, 10 µl of the CellLight BacMam MitoGFP reagent (40 particles per cell) for mitochondria matrix labeling (PDHA1–GFP) was added to each well, and incubation was continued for 16–20 h. On the day of the experiment, the cells were treated with 700 μl of a 200 nM solution of MAO-N_3_ (diluted from a 0.2 mM DMSO stock solution in Opti-MEM ph(–) buffer) for 1 h. The cells were washed once with Opti-MEM ph (–) and treated with 700 μl of a 100 nM solution of SiR-DBCO (diluted from a 2 mM DMSO stock solution into Opti-MEM ph(–) buffer) for 1 h. The cells were then washed with 700 µl of DMEM supplemented with 10% FBS and incubated in the same buffer for 1 h. For the experiment with TMRM, the cells were treated with 700 μl of a 100 nM solution of TMRM (diluted from a 0.1 mM DMSO stock solution into Opti-MEM ph(–) buffer) for 30 min, washed three times with 700 μl of DMEM supplemented with 10% FBS and incubated in the same buffer for 1 h. Finally, the cells were washed again with 700 μl of DMEM ph(–) with 10% FBS and imaged in the same buffer at 37 °C and 5% CO_2_. For depolarized cells, after labeling with MAO-SiR or TMRM, the cells were incubated with 700 μl of 10 μM FCCP in DMEM ph(–) with 10% FBS for 30 min, washed three times with 700 μl of DMEM ph(–) with 10% FBS and imaged in the same buffer at 37 °C and 5% CO_2_.

After imaging, cells were washed, trypsinized and pelleted at 200*g* for 3 min as described above. The pellet was resuspended in 100 µl of DPBS and transferred to a 1.5-ml microcentrifuge tube. Flow cytometry measurements were performed at room temperature with an Attune NxT flow cytometer. Hoechst 33342 was excited with a laser at 405 nm, and the emission filter was set at 440 ± 50 nm. The fluorophore TMRM was excited with a laser at 561 nm, and the emission filter was set at 585 ± 16 nm. MAO-SiR was excited with a laser at 638 nm, and the emission filter was set at 652 ± 10 nm. The collected data were analyzed using GraphPad Prism 8.4.3.

### Two-color structured illumination microscopy imaging of mitochondrial membranes using mEmerald–TOMM20 and MAO-SiR

Two days before imaging, HeLa cells were plated (2.5 × 10^4^ cells per well) in a four-well chamber and incubated overnight. After 16 h, the cells were transfected with plasmids encoding mEmerald–TOMM20 using FuGENE HD transfection reagent (Promega), according to manufacturer’s protocol, and incubated overnight. The following day, the cells were labeled with 600 μl of 75 nM MAO-N_3_ in DMEM ph(–) for 1 h, washed three times with 600 μl of DMEM ph(–), incubated with 600 μl of 0.6 μM SiR-DBCO in DMEM ph(–) for 1 h and washed again (six times for 10 min each with DMEM ph(–) supplemented with 10% FBS). The sample was subsequently visualized via Lattice SIM imaging on a Zeiss Elyra 7 microscope. The following parameters were used: laser powers of 6% (642 nm, estimated at 0.04 kW cm^–2^) and 3% (488 nm, estimated at 0.04 kW cm^–2^), field of view of 80.14 μm × 80.14 μm, camera exposure time of 40 ms, acquisition of 13 phases for SIM reconstruction, an LBF 405/488/561/642 beam splitter and a BP 495–550 + LP 655 filter set. Cells were incubated with DMEM ph(–) supplemented with 10% FBS at 37 °C and 5% CO_2_. SIM reconstruction was performed with Zeiss Zen Black software. Nonlinear regression for Gaussian and Lorentzian fitting of peaks from line profiles was performed using OriginLab OriginPro 2022b and plotted using Graphpad Prism 8.4.3.

### Two-color structured illumination microscopy imaging of mitochondrial membranes using BacMam 2.0 MitoGFP and MAO-SiR

Two days before imaging, HeLa cells were plated (2.5 × 10^4^ cells per well) in a four-well chamber and incubated overnight. After 16 h, the cells were transfected with 10 μl of Celllight BacMam 2.0 MitoGFP for 8 h, washed three times with 600 μl of DMEM ph(–) supplemented with 10% FBS and incubated overnight. The following day, the cells were labeled with 600 μl of 75 nM HAO-N_3_ in DMEM ph(–) for 1 h at 37 °C with 5% CO_2_, washed three times with 600 μl of DMEM ph(–), incubated with 600 μl of 0.6 μM SiR-DBCO in DMEM ph(–) for 1 h at 37 °C with 5% CO_2_ and washed again six times with DMEM ph(–) and 10% FBS for 10 min each. The sample was subsequently visualized via Lattice SIM imaging. The following parameters were used: laser powers of 6% (642 nm, estimated at 0.04 kW cm^–2^) and 3% (488 nm, estimated at 0.04 kW cm^–2^), field of view of 80.14 μm × 80.14 μm, camera exposure time of 40 ms, acquisition of 13 phases for SIM reconstruction, an LBF 405/488/561/642 beam splitter and a BP 495–550 + LP 655 filter set. Cells were incubated with DMEM ph(–) supplemented with 10% FBS at 37 °C and 5% CO_2_. SIM reconstruction was performed with Zeiss Zen Black software. Nonlinear regression for Gaussian and Lorentzian fitting of peaks from line profiles was performed using OriginLab OriginPro 2022b and plotted using Graphpad Prism 8.4.3.

### Time-lapse structured illumination microscopy imaging of the inner mitochondrial membrane using MAO-SiR

HeLa cells were plated (3.0 × 10^4^ cells per well) in four-well LabTek II chambers (Nunc, Thermo Fisher Scientific) and incubated overnight. The following day, the cells were labeled with 600 μl of 75 nM HAO-N_3_ in DMEM ph(–) for 1 h at 37 °C, washed three times with 600 μl of DMEM ph(–), incubated with 600 μl of 0.6 μM SiR-DBCO in DMEM ph(–) for 1 h at 37 °C and 5% CO_2_ and washed again six times with DMEM ph(–) and 10% FBS for 10 min each. Time-lapse SIM imaging of labeled cells was performed on a Zeiss Elyra 7 microscope using Zeiss Zen Black software. The following settings were used: laser power of 0.4% (642 nm, estimated at 0.0028 kW cm^–2^), field of view of 80.14 μm × 80.14 μm, camera exposure time of 40 ms, acquisition of 13 phases per frame for SIM reconstruction, intervals of 100 ms between each frame, acquisition of 500 frames in total over 297 s, an LBF 405/488/561/642 beam splitter and an SBS LP 560 filter set. SIM reconstruction was performed with Zeiss Zen Black software, and cells were incubated with DMEM ph(–) supplemented with 10% FBS at 37 °C and 5% CO_2_. Kymographs of line profiles (Fig. 4g) were generated using the KymographBuilder plugin in Fiji. Fluorescence intensity was obtained from randomly picked 3 μm × 3 μm regions using ImageJ and plotted using Graphpad Prism 8.4.3. The signal intensity half-life was determined by performing a nonlinear regression of the exponential decay function using OriginLab OriginPro 2022b.

### Time-lapse structured illumination microscopy imaging of the outer mitochondrial membrane using mEmerald–TOMM20

Two days before imaging, HeLa cells were plated (2.5 × 10^4^ cells per well) in a four-well chamber and incubated overnight. After 16 h, the cells were transfected with plasmids encoding mEmerald–TOMM20 using FuGENE HD transfection reagent (Promega), according to manufacturer’s protocol, and incubated overnight. The following day, the cells were visualized via Lattice SIM imaging on a Zeiss Elyra 7 microscope. The following settings were used: laser power of 0.4% (488 nm, estimated at 0.005 kW cm^–2^), field of view of 80.14 μm × 80.14 μm, camera exposure time of 40 ms, acquisition of 13 phases per frame for SIM reconstruction, interval time of 100 ms between each frame, acquisition of 500 frames in total over 297 s, an LBF 405/488/561/642 splitter and an SBS LP 560 filter set. Cells were incubated in DMEM ph(–) supplemented with 10% FBS at 37 °C and 5% CO_2_. SIM reconstruction was performed with Zeiss Zen Black software. Fluorescence intensity was obtained from randomly picked 3 μm × 3 μm regions using ImageJ and plotted using GraphPad Prism 8.4.3. The signal intensity half-life was obtained by performing a nonlinear regression of the exponential decay function using OriginLab OriginPro 2022b.

### Time-lapse structured illumination microscopy imaging of the outer mitochondrial membrane using Halo-TOMM20 and SiR-CA

Two days before imaging, HeLa cells were plated (2.5 × 10^4^ cells per well) in a four-well chamber and incubated overnight. After 16 h, the cells were transfected with plasmids encoding Halo-TOMM20 using FuGENE HD transfection reagent (Promega), according to manufacturer’s protocol, and incubated overnight. The following day, the cells were incubated with 600 μl of 2 μM SiR-CA in DMEM ph(–) for 1 h at 37 °C and CO_2_ and washed three times with 600 μl of DMEM ph(–) supplemented with 10% FBS. Cells were visualized via Lattice SIM imaging on a Zeiss Elyra 7 microscope. The following settings were used: laser power of 0.4% (488 nm, estimated at 0.005 kW cm^–2^), field of view of 80.14 μm × 80.14 μm, camera exposure time of 40 ms, acquisition of 13 phases per frame for SIM reconstruction, interval time of 100 ms between each frame, acquisition of 500 frames over 297 s, an LBF 405/488/561/642 beam splitter and an SBS LP 560 filter set. Cells were incubated with DMEM ph(–) supplemented with 10% FBS at 37 °C and 5% CO_2_. SIM reconstruction was performed with Zeiss Zen Black software. Fluorescence intensity was obtained from randomly picked 3 μm × 3 μm regions using ImageJ and plotted using Graphpad Prism 8.4.3. The signal intensity half-life was obtained by performing a nonlinear regression of the exponential decay function using OriginLab OriginPro 2022b.

### Single-molecule localization microscopy imaging of inner mitochondrial membrane structure using HMDS_655_-DBCO and MAO-N_3_

Before seeding, four-well LabTek II (Nunc) chambers were treated by sonication for 15 min in 1 M KOH, rinsed three times with deionized water, sterilized with 100% ethanol and air dried overnight in a biological safety cabinet. HeLa cells were seeded onto pretreated plates at a density of 3.0 × 10^4^ cells per well and incubated overnight at 37 °C with 5% CO_2_. The following day, the cells were labeled with 600 μl of 75 nM MAO-N_3_ in DMEM ph(–) for 40 min at 37 °C and 5% CO_2_, washed three times with 600 μl of DMEM ph(–), incubated with 600 μl of 0.6 μM HMDS_655_-DBCO in DMEM ph(–) for 20 min at 37 °C and 5% CO_2_ and washed again six times with DMEM ph(–) and 10% FBS for 10 min each at 37 °C and 5% CO_2_. Finally, the medium was exchanged with 600 μl of DMEM ph(–) supplemented with 1% FBS, 100 μM Trolox and 500 μM sodium ascorbate. The sample was subsequently visualized via SMLM imaging on an inverted Zeiss Elyra 7 microscope equipped with a Plan-Apo ×63/1.4-NA oil immersion lens (40% laser power for the 642-nm laser, 60.57° TIRF mirror angle, TIRF-uHP mode, 2.5-ms exposure time for each frame and 6,000 frames obtained in 36 s).

### Stimulated emission depletion imaging of cristae structures and their dynamics using SiR-DBCO and MAO-N_3_

HeLa cells were plated (2.2 × 10^4^ cells per well) in a four-well, number 1.5 glass-bottom chamber (Ibidi, 80827) and incubated for 42 h. On the day of imaging, the cells were labeled with 700 μl of 200 nM MAO-N_3_ in Opti-MEM ph(–) for 1 h, washed once with 700 μl of DMEM ph(–), incubated with 700 μl of 100 nM SiR-DBCO in Opti-MEM ph(–) for 1 h, washed with 700 μl of DMEM supplemented with 10% FBS and incubated for 1 h. The medium was exchanged with DMEM ph(–), and the sample was subsequently visualized via STED imaging (20% excitation laser power, depleted by 24% STED laser power). Cells were incubated in DMEM ph(–) at 37 °C and 5% CO_2_. Deconvolution of gated STED images was performed based on the theoretical point spread function and the classic maximum likelihood estimation^54^ method using the commercial software package SVI Huygens Pro Imaging parameters for time lapse (256 × 128, 1.3-s time interval, total of 125 frames, scan rate of 100 Hz, no line accumulation/average, pixel size of 30 nm).

### Reporting summary

Further information on research design is available in the Nature Portfolio Reporting Summary linked to this article.

## Online content

Any methods, additional references, Nature Portfolio reporting summaries, source data, extended data, supplementary information, acknowledgements, peer review information; details of author contributions and competing interests; and statements of data and code availability are available at 10.1038/s41589-023-01450-y.

### Supplementary information

Supplementary Information Supplementary Figs. 1–5 and Note (synthesis of HAO-N_3_, MAO-N_3_ and HMDS_655_-DBCO and in vitro characterization of MAO-N_3_ and HAO-N_3_).

Reporting Summary

Supplementary Video 1 Time-lapse images of hiPSC-CMs labeled with MAO-SiR. hiPSC-CMs were labeled with MAO-N_3_ and SiR-DBCO (Methods) and imaged using a point-scanning confocal microscope (*n* = 213 frames; *t* = 110 s).

Supplementary Video 2 Overnight time-lapse confocal imaging of HeLa cells labeled with MAO-SiR. HeLa cells were labeled with MAO-N_3_ and SiR-DBCO (Methods) and imaged using a point-scanning confocal microscope (*n* = 220 frames; *t* = 12.5 h).

Supplementary Video 3 Overnight brightfield time-lapse imaging of untreated HeLa cells. HeLa cells in blank medium were imaged using a point-scanning confocal microcope as described in the Methods (*n* = 220 frames; *t* = 12.5 h).

Supplementary Video 4 Overnight time-lapse confocal imaging of HeLa cells labeled with MitoTracker Deep Red. HeLa cells were labeled with MitoTracker Deep Red (Methods) and imaged using a point-scanning confocal microscope (*n* = 220 frames; *t* = 12.5 h).

Supplementary Video 5 Time-lapse lattice SIM imaging of mitochondria labeled with MAO-SiR. HeLa cells were labeled with MAO-N_3_ and SiR-DBCO (Methods) and imaged using a widefield microscope equipped with lattice SIM (*n* = 500 frames; *t* = 297 s).

Supplementary Video 6 Time-lapse lattice SIM imaging of mitochondria labeled with mEmerald–TOMM20. HeLa cells expressing the OMM GFP marker mEmerald–TOMM20 were imaged using a widefield microscope equipped with lattice SIM (Methods; *n* = 500 frames; *t* = 297 s).

Supplementary Video 7 Time-lapse lattice SIM imaging of mitochondria labeled with Halo-TOMM20 and SiR-CA. HeLa cells expressing the OMM marker Halo-TOMM20 were labeled with SiR-CA (Methods) and imaged using a widefield microscope equipped with lattice SIM (*n* = 500 frames; *t* = 297 s).

Supplementary Video 8 Time-lapse STED imaging of mitochondria labeled with MAO-SiR. HeLa cells were labeled with MAO-N_3_ and SiR-DBCO (Methods) and imaged using a point-scanning confocal microscope equipped with a 775-nm STED laser (*n* = 125 frames; *t* = 162.5 s).

Supplementary Video 9 Time-lapse STED imaging of mitochondria labeled with SiR-CA and Halo-TOMM20. HeLa cells expressing the OMM marker Halo-TOMM20 were labeled with SiR-CA (Methods) and imaged using a point-scanning confocal microscope equipped with a 775-nm STED laser (*n* = 125 frames; *t* = 162.5 s).

Supplementary Video 10 Time-lapse STED imaging of mitochondria labeled with SiR-SNAP and COX8A-SNAP. HeLa cells expressing the OMM marker COX8A-SNAP were labeled with SiR-SNAP (Methods) and imaged using a point-scanning confocal microscope equipped with a 775-nm STED laser (*n* = 125 frames; *t* = 162.5 s).

Supplementary Data 1 Source data for Supplementary Fig. 2.

Supplementary Data 2 Source data for Supplementary Fig. 3.

Supplementary Data 3 Source data for Supplementary Fig. 4.

Supplementary Data 4 Source data for Supplementary Fig. 5.

### Source data

Source Data Fig. 2 Normalized UV–Vis spectra and LC–MS trace statistical source data.

Source Data Fig. 3 Statistical source data for confocal fluorescent signal decay and cell proliferation rate.

Source Data Fig. 4 Source data for line profiles and SIM fluorescent signal decay.

Source Data Fig. 5 Source data for SMLM line profiles.

Source Data Fig. 6 Source data for STED line profiles and fluorescent signal decay.

Source Data Extended Data Fig. 2 UV–Vis absorption and fluorescent emission and extinction coefficient plot of HAO-N_3_ and MAO-N_3_.

Source Data Extended Data Fig. 3 Statistical source data for cell viability and real-time respirometry assays.

Source Data Extended Data Fig. 4 Statistical source data for fluorescent signal colocalization.

Source Data Extended Data Fig. 5 Statistical source data for fluorescent signal colocalization.

Source Data Extended Data Fig. 6 Statistical source data for confocal fluorescent signal colocalization, intensity and flow cytometry.

Source Data Extended Data Fig. 8 Statistical source data for SIM fluorescent signal decay.

Source Data Extended Data Fig. 9 Source data for STED and corresponding confocal line profiles.

## Data Availability

All data supporting the findings of this study are available within the paper and Supplementary Information files. The unprocessed microscopy data are not deposited due to their large size and are available upon reasonable request from the corresponding author. Source data are provided with this paper.
