# Supplementary material for: Long-term super-resolution inner mitochondrial membrane imaging with a lipid probe
Source: Nat Chem Biol. 2023 Oct 19;20(1):83–92. doi: 10.1038/s41589-023-01450-y (PMC10746544; doi:10.1038/s41589-023-01450-y)
Supplement: Supplementary file 1 — Supplementary Figs. 1–5 and Note (synthesis of HAO-N3, MAO-N3 and HMDS655-DBCO and in vitro characterization of MAO-N3 and HAO-N3). [file 41589_2023_1450_MOESM1_ESM.pdf]

nature chemical biology

Article

https://doi.org/10.1038/s41589-023-01450-y

Long-term super-resolution inner mitochondrial membrane imaging with a lipid probe

In the format provided by the
authors and unedited

Table of Contents

I. Supplementary Figure

II. Supplementary Note: synthesis of MAO-N_3 (**1**), HAO-N_3 (**2**), and $HMDS_{655}$-DBCO (**4**)

   A. General Considerations

   B. Synthesis of MAO-N_3 (**1**)

   C. Synthesis of HAO-N_3 (**2**)

   D. Synthesis of $HMDS_{655}$-DBCO (**4**)

III. Supplementary Note: in vitro characterization of MAO-N_3 and HAO-N_3

   A. In vitro spectroscopic characterization

   B. In vitro SPAAC reaction

   C. Media exchange for HeLa cells in galactose supplemented media

   D. Cell viability assays

   E. Real-time cell respirometry

IV. NMR Spectra

V. References

I. Supplementary Figure

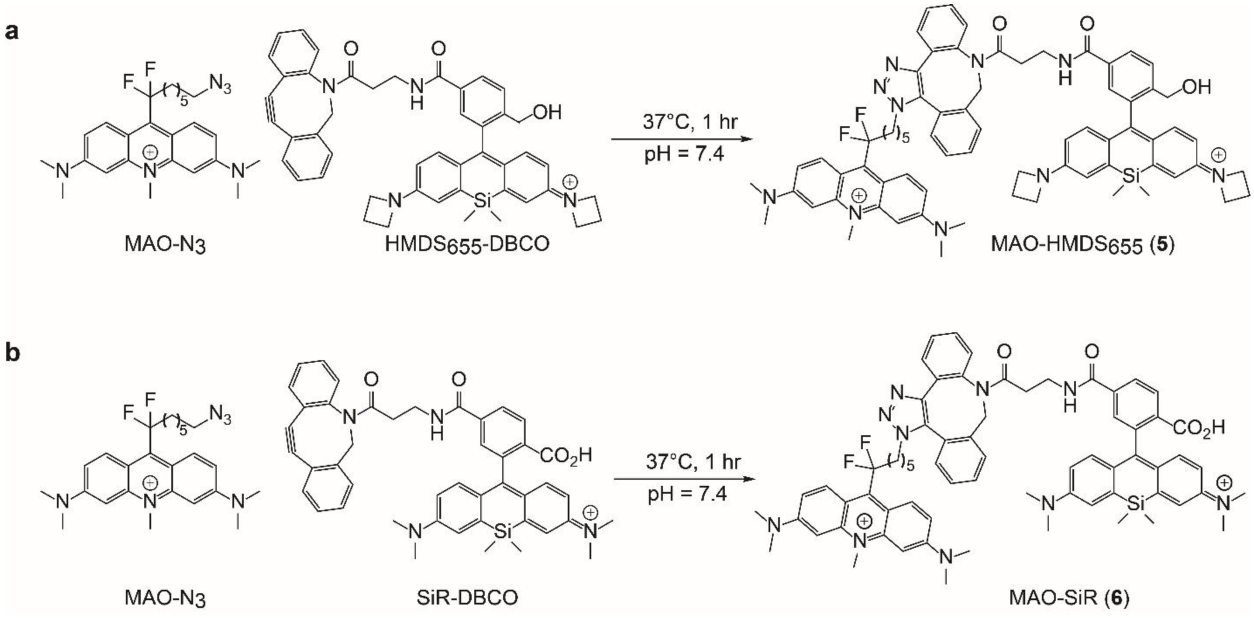

Supplementary Fig 1. Inner mitochondrial membrane-specific HIDE probes MAO-HMDS$_{655}$ and MAO-SiR assembled using SPAAC reaction.

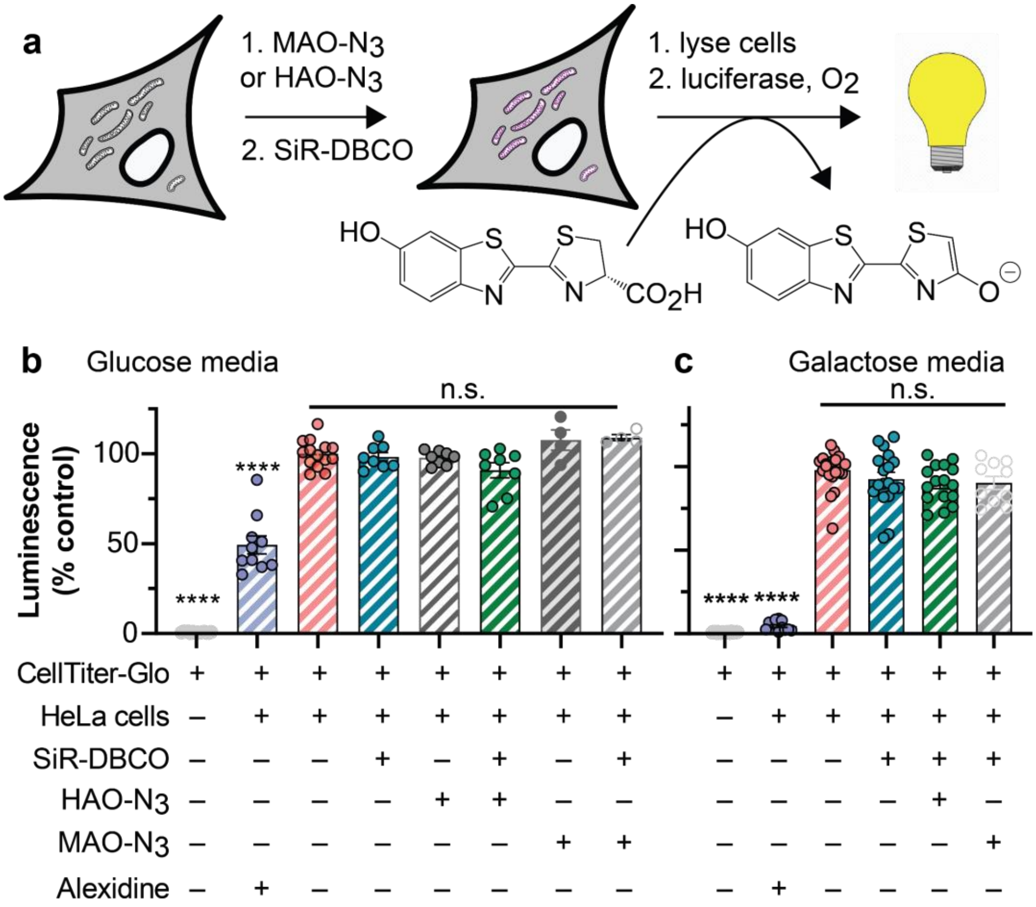

Supplementary Fig. 2. **The HIDE probes HAO-SiR and MAO-SiR generated using HAO-N_3 and MAO-N_3 are non-toxic, even in galactose supplemented media**. **a,** HeLa cells incubated in standard glucose (DMEM with 4.5 g/L glucose, supplemented with 10% FBS) or oxidative galactose-rich media (DMEM with 4.5 g/L galactose, supplemented with 10% FBS) were treated with HAO-N_3 or MAO-N_3 and SiR-DBCO and the ATP levels measured immediately as described in **Online Methods**. Plots show the relative bioluminescence signals (% relative to untreated cells). **b&c,** Plots show the relative bioluminescence signals of HeLa cell lysates incubated in **b,** standard or **c,** oxidative media. Untreated cells serve as a negative control; cells treated with 5 µM alexidine serve as a positive control. Alexidine inhibits the PTEN-like mitochondrial phosphatase PTPMT1 and disrupts mitochondrial integrity[29]. Error bars = s.e.m.; **** $p < 0.0001$, n.s. (not significant) $0.4180<p<1$ (for **b**) or $0.2082<p<1$ (for **c**) from one-way

ANOVA with Dunnett's post-analysis accounting comparison to the negative control where the cells were incubated with cell culture media only. Each bar represents the average of 12 biological replicates.

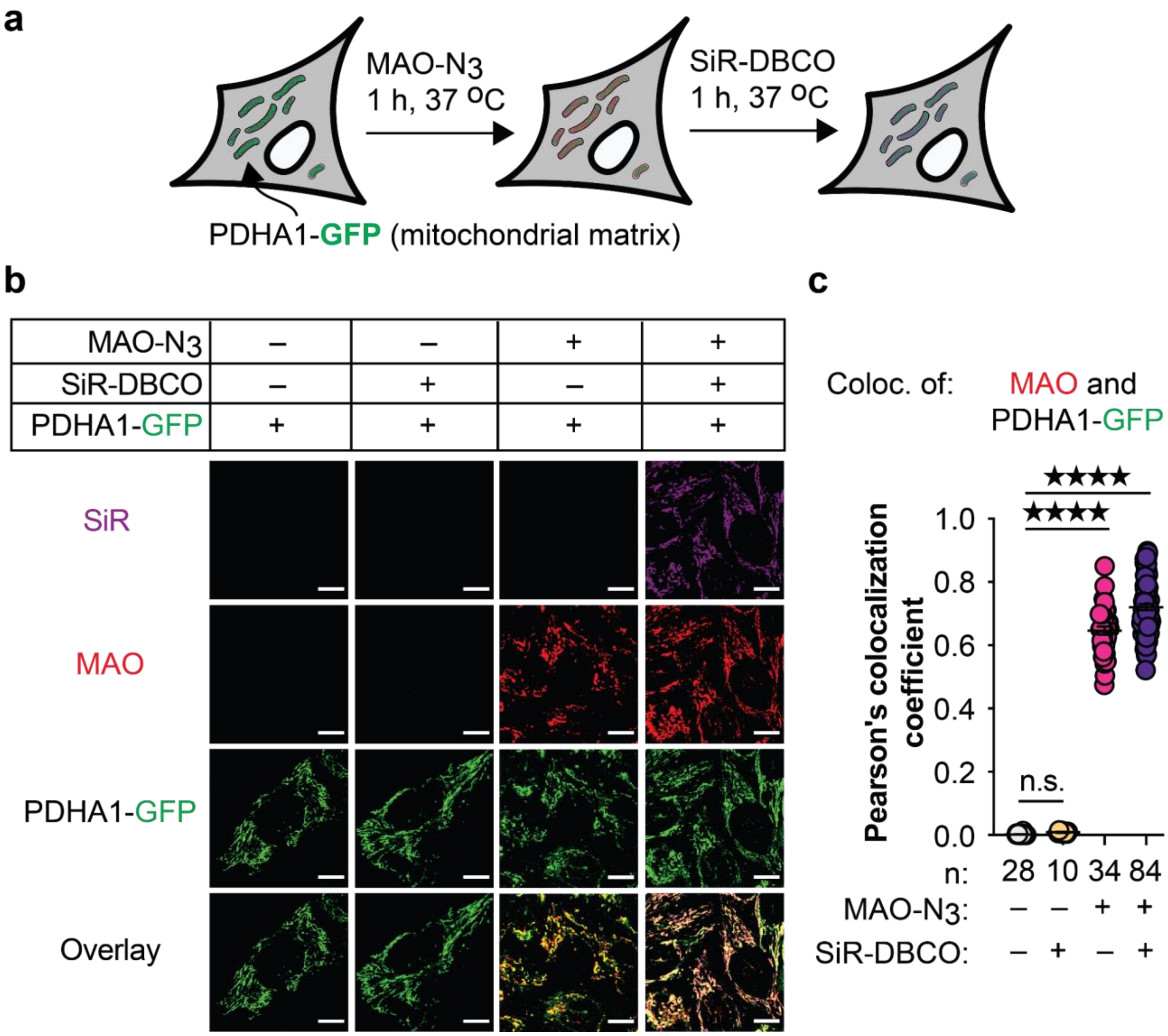

Supplementary Fig. 3. MAO-SiR, the HIDE probe generated from MAO-N_3 and SiR-DBCO, localizes to mitochondria. **a,** HeLa cells expressing the mitochondrial matrix marker PDHA1-GFP (CellLight™ Mitochondria-GFP, BacMam 2.0) were treated as described in **Online Methods**. Cells were visualized using a point-scanning confocal microscope. **b,** Confocal images representing the colocalization of signal from MAO or SiR with PDHA1-GFP. **c,** Pearson's colocalization coefficients PCC (MAO/PDHA1-GFP) = 0.77 ± 0.01. *n* = 28, 10, 34 or 84 cells for the conditions indicated in the graph, sourced from at least two biological replicates.

Scale bar: 10 µm. Error bars = s.e.m. **** p<0.0001, n.s. (not significant) p = 0.9890, from one-way ANOVA with Tukey's multiple comparison test.

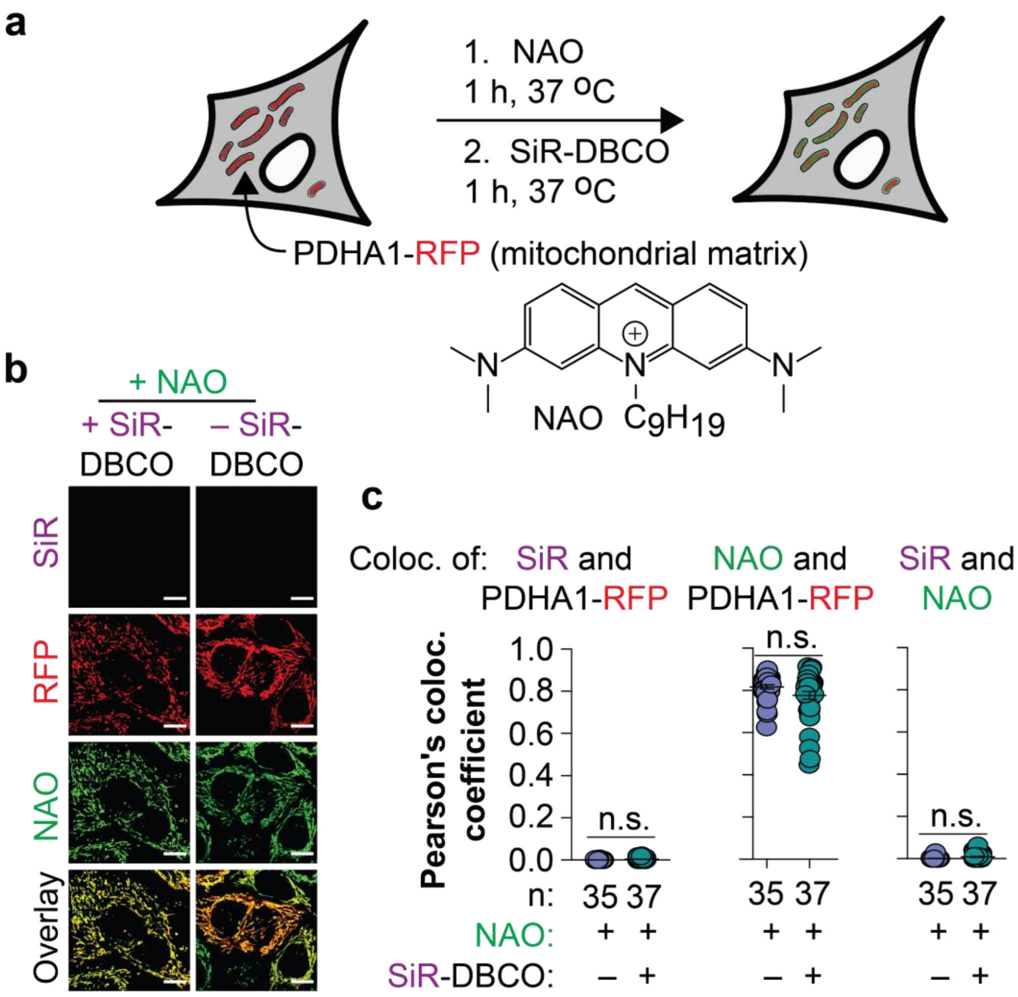

Supplementary Fig. 4. **The azido group of MAO-N_3 and HAO-N_3 recruits SiR-DBCO to the mitochondria. a,** HeLa cells expressing the mitochondrial matrix marker PDHA1-RFP (CellLight™ Mitochondria-RFP, BacMam 2.0) were treated with NAO and SiR-DBCO as described in **Online Methods** and visualized using a point-scanning confocal microscope. **b,** Representative confocal images used for quantification of Pearson's colocalization coefficients. **c,** Pearson's colocalization coefficients (PCC) representing the colocalization of signal from NAO or SiR with mitochondria marker PDHA1-RFP in HeLa cells. PCC (NAO/PDHA1-RFP) = 0.81 ± 0.01; no significant signal was observed in the SiR channel. Scale bar: 10 µm. n = # of cells. Error bars = s.e.m. n.s. (not significant) 0.2027<p<0.9991, from one-way ANOVA with Tukey's multiple comparison test.

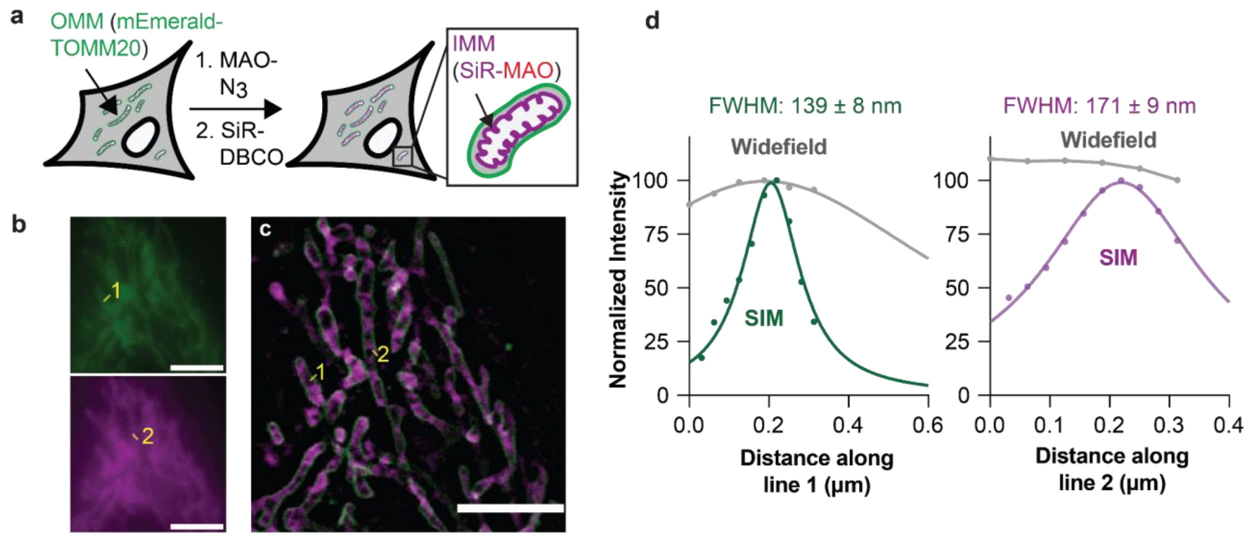

Supplementary Fig. 5. Two-color SIM imaging of mitochondria using the HIDE probe MAO-SiR and the OMM marker mEmerald-TOMM20. a, HeLa cells expressing mEmerald-TOMM20 were treated with MAO-N_3 and SiR-DBCO as described in **Online Methods** and imaged using lattice SIM. **b,** Widefield images of HeLa cells treated as described in **a**; mEmerald-TOMM20 (TOP, green, 510 nm) and SiR (BOTTOM, magenta, 660 nm) channels. Scale bar: 5 µm. **c,** The reconstructed SIM image showing the merger of SiR and mEmerald signals. **d,** Plot illustrating the fit of signals along line **1** (LEFT) in panels **b** and **c** to a Gaussian function to establish FWHM values of 139 ± 8 nm for the SIM image. Plot illustrating the fit of signals along line 2 (RIGHT) in panels **b** and **c** to a Gaussian function to establish a FWHM of 171 ± 9 nm for the SIM image. Images in **b-c** are representative for at least 3 biologically independent replicates. Same dataset as **Fig. 4b** was reused here to demonstrate resolution for consistency and ease of reading.

Supplementary Note

II. Synthesis of HAO-N_3, MAO-N_3 and HMDS$_{655}$-DBCO

A. General considerations

Unless otherwise noticed, all reactions were performed in a nitrogen atmosphere. Chemicals used for synthesis were either prepared according to literature reports or purchased from commercial sources and used without further purification. Flash chromatography was performed using a Teledyne Isco CombiFlash Rf system using pre-packed columns with RediSep Rf silica (40–60 µm). ^{1}H NMR and ^{13}C NMR spectra were recorded on Bruker Neo-500 (500 MHz for ^{1}H, 126 MHz for ^{13}C and 471 MHz for ^{19}F) or AV-600 (600 MHz for ^{1}H and 151 MHz for ^{13}C) NMR spectrometers using Bruker Topsin 3.6 and analyzed by Mestrelab MestreNova 14.2. The values of chemical shifts (δ) are reported in ppm relative to the solvent residual signals of CD_3OD (3.31 ppm for ^{1}H, 49.0 ppm for ^{13}C), $CDCl_3$ (7.26 ppm for ^{1}H, 77.2 ppm for ^{13}C) or CD_3CN (1.94 ppm for ^{1}H, 1.32, 118.3 ppm for ^{13}C). Coupling constants (J) are reported in Hz. High-resolution mass spectra (HRMS) were recorded on an Angilent QTOF LCMS with ESI connected to an Agilent UHPLC. HPLC purifications were performed on a reverse-phase column (YMC-Triart C18, 150 mm × 10 mm S-5 µm, 12 nm) using an Angilent HPLC system.

B. Synthesis of MAO-N_3 (1)

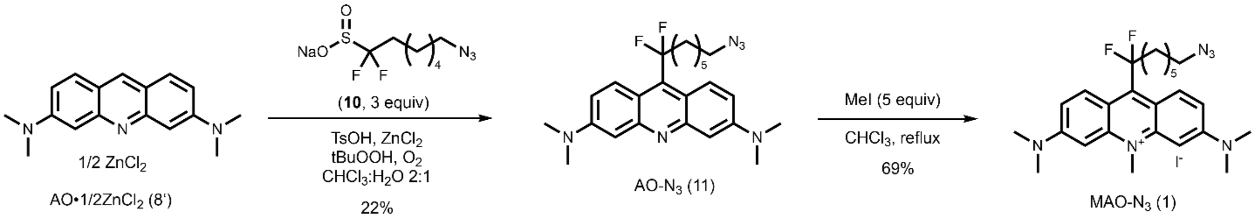

Preparation of 9-(7-azido-1,1-difluoroheptyl)-*N*^{3},*N*^{3},*N*^{6},*N*^{6}-tetramethylacridine-3,6-diamine (11). AO-N_3 (**11**) was synthesized as reported[2]. Difluoride **10** (90.0 mg, 0.34 mmol, 4.0 equiv)[2], acridine orange hemi $ZnCl_2$ salt (34.8 mg, 0.09 mmol, 1.0 equiv) and $ZnCl_2$ (17.5 mg, 0.13 mmol, 1.5 equiv) was dissolved in $CHCl_3$ (1 mL) and H_2O (0.5 mL) in a 4 mL vial equipped with septa screw cap, followed by addition of TsOH•H_2O (32.5 mg, 0.17 mmol, 2 equiv). The reaction mixture was cooled to 0 ℃ and *tert*-butyl hydroperoxide (TBHP, 70% in water, 0.07 ml, 60.5 mg, 0.47 mmol, 5.5 equiv) was added dropwise with vigorous stirring over 5 min. The reaction was then heated at 50 °C for 1 h, cooled to RT, and stirred for 24 h under continuous air flow. The mixture was then diluted with CH_2Cl_2 (2 mL), separated, and the aqueous layer was washed with CH_2Cl_2 (2 x 2 mL). The combined organic layers were washed with $NaHCO_3$, brine, dried

(Na_2SO_4), and concentrated *in vacuo*. AO-N_3 (**11**) was obtained as a deep-red sticky solid (10 mg, 22%), upon purification by flash column chromatography (neutral Al_2O_3, 0 to 5% MeOH:CH_2Cl_2). The NMR spectrum of the obtained product matched the literature report.

Preparation of MAO-N_3 (9-(7-azido-1,1-difluoroheptyl)-3,6-bis(dimethylamino)-10-methylacridin-10-ium, 1). To an 8 mL microwave vial was added a $CHCl_3$ (3 mL) solution of **3** (10 mg, 0.02 mmol, 1.0 equiv) and then CH_3I (32.2 mg, 0.23 mmol, 10 equiv). The reaction mixture was heated to reflux overnight before cooling and purification *via* flash column chromatography (neutral Al_2O_3, 0 to 5% MeOH:CH_2Cl_2). The corresponding product **MAO-N_3** (**1**) was obtained as a deep red solid (9.1 mg, 69%). ^1H NMR (600 MHz, Chloroform-*d*) δ 8.20 (d, J = 9.9 Hz, 2H), 7.12 (dd, J = 10.0, 2.5 Hz, 2H), 6.89 (d, J = 2.5 Hz, 2H), 4.54 (s, 3H), 3.41 (s, 12H), 3.30 (t, J = 6.8 Hz, 2H), 2.52 (tt, J = 17.1, 8.2 Hz, 2H), 1.83 – 1.74 (m, 2H), 1.63 (tt, J = 7.1, 6.8 Hz, 2H), 1.53 – 1.42 (m, 4H). ^{13}C NMR (151 MHz, Chloroform-*d*) δ 154.4, 145.3 (t, J = 24.3 Hz), 144.6, 129.6 (t, J = 10.2 Hz), 125.1 (t, J = 247.4 Hz), 115.0, 114.5, 94.7, 51.4, 41.4, 41.3, 40.6 (t, J = 24.6 Hz), 28.8, 28.7, 26.6, 22.1. ^{19}F NMR (376 MHz, Chloroform-*d*) δ -81.48 (t, J = 17.4 Hz). HRMS (ES+) calcd for ($C_{23}H_{33}F_2N_6$) [M^+] 455.2729, found 455.2719.

C. Synthesis of HAO-N_3 (2)

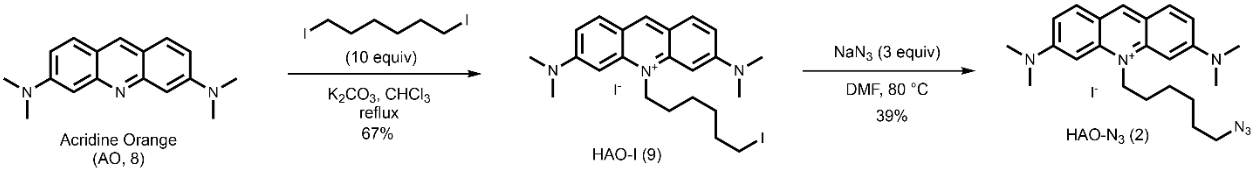

Acridine orange “free base” (**8**) was obtained according to literature[1]. Briefly, 4 g acridine orange ½ $ZnCl_2$ (Chem-Impex) was dissolved 100 mL H_2O and then treated with excess NaOH solution (4 N) for 30 min. The free acridine orange was extracted with CH_2Cl_2, washed with water, and dried over sodium sulfate. Removal of solvent under reduced pressure and drying under vacuum afforded 1.85 g of free acridine orange as a dark red-brown powder, which was used in the next step without further purification.

Preparation of 3,6-bis(dimethylamino)-10-(6-iodohexyl)acridin-10-ium (9). To a 20 mL microwave vial charged with K_2CO_3 (17.25 mg, 0.125 mmol, 0.5 equiv) was added acridine orange (**8**) solution (92.5 mg, 0.25 mmol, 1 equiv in 5 mL $CHCl_3$), followed by addition of 1,6-diiodohexane (0.41 mL, 2.5 mmol, 10 equiv). The reaction mixture was heated at reflux for 16h, cooled, filtered, and concentrated *in vacuo*. The product **9** was obtained as a dark-red solid (100 mg, 67%), upon purification by flash column chromatography (neutral Al_2O_3, 0 to 5%

MeOH:CH_2Cl_2). ^{1}H NMR (500 MHz, $CDCl_3$) δ 8.68 (s, 1H), 7.89 (d, *J* = 9.3 Hz, 2H), 7.05 (dd, *J* = 9.3, 2.1 Hz, 2H), 6.67 (d, *J* = 2.2 Hz, 2H), 4.99 – 4.89 (m, 2H), 3.25 (t, *J* = 6.8 Hz, 2H), 2.01 (p, *J* = 7.8 Hz, 2H), 1.93 – 1.82 (m, 2H), 1.80 – 1.70 (m, 2H), 1.63 – 1.58 (m, 2H). ^{13}C NMR (126 MHz, $CDCl_3$) δ 155.7, 143.1, 142.8, 133.4, 117.3, 114.2, 93.2, 48.5, 41.5, 33.1, 30.5, 26.3, 26.2, 7.9. HRMS (ES+) calcd for ($C_{23}H_{31}IN_3$) [M^+] 476.1557, found 476.1549.

Preparation of HAO-N_3. Compound **9** (30.2 mg, 0.05 mmol, 1.0 equiv) and NaN_3 (9.8 mg, 3 equiv) were dissolved in DMF (1 mL) in an 8 mL microwave vial. The reaction was heated at 80 ℃ for 16 h and monitored by TLC. Upon completion, the reaction mixture was diluted with 30 mL CH_2Cl_2 and washed 3X with brine (30 mL), dried (Na_2SO_4) and evaporated. The corresponding product HAO-N_3 (**1**) was obtained as a dark-red solid (10.2 mg, 39%), upon purification by flash column chromatography (neutral Al_2O_3, 0 to 10% MeOH:CH_2Cl_2). ^{1}H NMR (500 MHz, Chloroform-*d*) δ 8.64 (s, 1H), 7.90 (d, *J* = 9.3 Hz, 2H), 7.10 (dd, *J* = 9.2, 2.2 Hz, 2H), 6.78 (d, *J* = 2.3 Hz, 2H), 5.10 – 4.94 (m, 2H), 3.36 (s, 12H), 3.31 (t, *J* = 6.7 Hz, 2H), 2.03 (tt, *J* = 7.9 Hz, 2H), 1.76 (tt, *J* = 7.6 Hz, 2H), 1.71 – 1.64 (m, 2H), 1.59 – 1.52 (m, 1H). ^{13}C NMR (126 MHz, $CDCl_3$) δ 155.8, 143.3, 142.8, 133.5, 117.3, 114.2, 93.0, 51.5, 47.9, 41.1, 29.7, 28.9, 26.8, 26.3. HRMS (ES+) calcd for ($C_{23}H_{31}N_6$) [M^+] 391.2605, found 391.2594.

D. Synthesis of HMDS$_{655}$-DBCO (4)

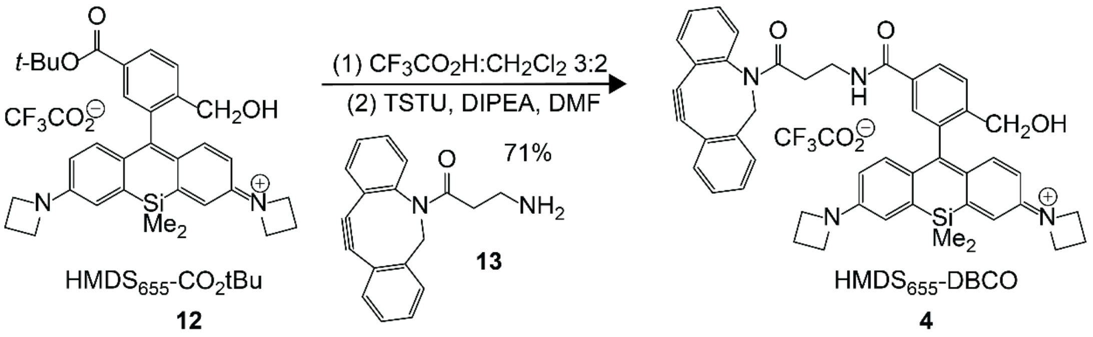

Preparation of HMDS$_{655}$-CO_2tBu 12. Compound **12** was synthesized according to a previous report[3].

Preparation of HMDS$_{655}$-DBCO (4). To a 4 mL vial equipped with a septa screw cap was added **12** (11.6 mg, 0.02 mmol, 1 equiv) and 2 mL of a 3:2 mixture of CF_3CO_2H and CH_2Cl_2. The resulting reaction mixture was stirred for 2 h and monitored by TLC. Upon completion, the mixture was concentrated *in vacuo* and thoroughly dried under high vacuum. The product was redissolved in DMF (2 mL), DIPEA (13.9 mg, 0.11 mmol, 5.0 equiv) was added, and the reaction mixture stirred for 5 mins. A solution of **13** (17.8 mg, 0.06 mmol, 3.0 equiv), TSTU (8.1 mg, 0.03

mmol, 1.25 equiv) and DIPEA (13.9 mg, 0.11 mmol, 5.0 equiv) in DMF (1 mL) was then added. The reaction was stirred overnight and purified using an Agilent semi-prep HPLC system on a reverse-phase column (YMC-Triart C18, 150 mm × 10 mm S-5 μm, 12 nm) with eluents A (H_2O with 1% TFA) and B(CH_3CN with 1% TFA). The corresponding product $HMDS_{655}$-DBCO (**4**) was obtained as a blue solid (10.9 mg, 71%). ^1H NMR (500 MHz, MeOD) δ 7.64 (d, *J* = 7.8 Hz, 1H), 7.58 (s, 1H), 7.49 – 7.39 (m, 4H), 7.32 – 7.26 (m, 2H), 7.12 – 7.09 (m, 1H), 7.08 – 7.04 (m, 2H), 6.82 – 6.77 (m, 2H), 6.71 (dd, *J* = 8.1, 2.7 Hz, 2H), 6.44 (ddd, *J* = 8.6, 5.8, 2.6 Hz, 2H), 5.42 (s, 2H), 5.14 (d, *J* = 14.0 Hz, 2H), 3.92 – 3.79 (m, 8H), 3.69 (s, 2H), 2.48 (dq, *J* = 17.2, 5.9 Hz, 2H), 2.40 – 2.27 (m, 4H), 0.60 (s, 3H), 0.48 (s, 3H). ^{13}C NMR (151 MHz, MeOD) δ 173.48, 169.46, 152.59, 152.41, 152.30, 152.25, 149.33, 140.71, 140.61, 139.24, 135.19, 134.66, 133.34, 130.35, 129.92, 129.68, 129.62, 129.02, 128.82, 128.16, 127.77, 126.56, 124.53, 124.20, 123.65, 121.76, 119.23, 117.29, 116.49, 115.61, 115.34, 114.55, 114.50, 108.88, 94.23, 74.10, 56.51, 53.69, 53.66, 37.43, 35.36, 17.91, 0.15, -0.18.HRMS (ES+) calcd for ($C_{47}H_{45}N_4O_3Si^+$) [M^+] 741.3255, found 741.3261.

III. Supplementary Note: in vitro characterization of MAO-N_3 and HAO-N_3

A. In vitro spectroscopic characterization.

Solutions used for absorption and emission spectra and quantum yield were obtained by diluting a 2 mM DMSO stock solution of each fluorophore with Dulbecco's phosphate-buffered saline (DPBS, pH 7.4) to the desired concentration (2 μM to 125 nM) . Absorption spectra were measured at room temperature using a Perkin-Elmer Lambda365 spectrometer in 1 cm pathlength, 1 mL cuvettes. Extinction coefficients were determined by performing a linear fitting of absorbance maxima (490 nm for HAO-N_3, 520 nm for MAO-N_3) against concentration of MAO-N_3 or HAO-N_3. Fluorescence spectra were measured at room temperature using a Jobin Yvon Fluoromax fluorimeter using 1 cm pathlength, 3.5 mL cuvettes. Absolute quantum yield was measured on Hamamatsu Quantaurus-QY (model No. C13534) using 1 cm pathlength, 3.5 mL cuvettes.

B. In vitro SPAAC reaction.

In a 300 μL LCMS vial (equipped with high recovery insert and pre-slit rubber septum), 100 μL MAO-N_3 (100 μM) and 100 μL of $HMDS_{655}$-DBCO (400 μM) solutions in DI H_2O were mixed and incubated at 37 ^oC. As is indicated in **Fig. 2d**, 1 μL aliquots of the reaction mixture were withdrawn upon mixing, after 15 min and 30 min of incubation, respectively. The aliquots were

analyzed by a Waters Acquity SQD2 UPLC-MS system equipped with a 2.1 mm x 30 mm, CSH C18 column with eluents A (H_2O with 0.1% HCO_2H) and B (CH_3CN with 0.1% HCO_2H).

C. Media exchange for HeLa cells in galactose supplemented media.

Five days before the experiment, HeLa cells were seeded in a T-75 tissue culture treated flask at a density of 1.0×10^6 cells and incubated in DMEM supplemented with 10% FBS and 4.5 g/L glucose at 37 °C, 5% CO_2 for 24 h. On day 2, the media was exchanged with DMEM supplemented with 10% FBS, 2.25 g/L glucose and 2.25 g/L galactose and incubated for another 24h. On day 3, the media was exchanged to DMEM, 10% FBS, 1.125 g/L glucose and 3.375 g/L galactose and to media supplemented with 4.5 g/L galactose on day 4. 24 h after the final media exchange step, the HeLa cells were plated for cell viability test.

D. Cell viability assays.

HeLa cells were plated into the wells of a flat-bottom 96-well plate (2×10^4 cells per well in 300 µL of media) and each well was incubated overnight. On the following day, the cells in each well were treated with 300 µL DMEM (negative control) or DMEM solutions of 100 nM HAO-N_3, MAO-N_3, or 5 µM alexidine (cytotoxicity positive control) for 1 h. Each well was then washed twice with 300 µL DMEM ph(-), followed by a 1 h incubation with 300 µL of DMEM ph(-) solutions of either 750 nM $HMDS_{655}$-DBCO or 750 nM SiR-DBCO. The media in each well was then exchanged with 300 µL DMEM supplemented with 10% FBS and incubation continued for 20 min. The buffer exchange and incubation was repeated for a total of 3 times. The media in each well was discarded, replaced with 100 µL of DPBS, and 100 µL CellTiterGlo 2.0 (Promega) solution was added. The plate was incubated at room temperature for 15 min in the dark. The bioluminescent level of each well was measured using a Synergy H1 plate reader (96-well opaque-walled tissue culture plate, 22 ^oC). Each of the relative bioluminescence signals (% relative to untreated cells) shown represents the average of 12 replicates.

E. Real-time cell respirometry.

Oxygen consumption rates (OCR) and extracellular acidification rates (ECAR) were measured using a Seahorse XFe24 Extracellular Flux Analyzer (Agilent). HeLa cells ($6.5\text{-}8.5 \times 10^4$ cells per well) were seeded on the cell culture microplate (Agilent Technologies, 100777-004) and incubated overnight. On the following day, the cells in each well were treated with 500 µL DMEM (basal conditions) or DMEM solutions of 200 nM MAO-N_3, or 100 nM MitoTracker Deep Red (control) for 1 h. Each well was then washed twice with 500 µL DMEM ph(-), followed by a

1 h incubation with 500 µL of DMEM ph(-) media or DMEM ph(-) solutions of either 100 nM $HMDS_{655}$-DBCO or 100 nM SiR-DBCO. The media in each well was then exchanged with 500 µL DMEM supplemented with 10% FBS and incubation continued for 30 min. The buffer exchange and incubation was repeated for a total of 2 times. The media in each well was then exchanged 3 times with 500 µL DMEM $NaHCO_3$(-) supplemented with 10% FBS, 4.5 g/L glucose or galactose, 2 mM glutamine, and 1 mM sodium pyruvate, and equilibrated in a CO_2-free incubator at 37 ^{o}C for 1 h before measuring OCR and ECAR values at different metabolic states. Each well was treated with oligomycin (final concentration 1 µM), FCCP (final concentration 10 µM), antimycin A (final concentration 1 µM) and rotenone (final concentration 100 nM) to assess the electron transport chain function. OCR and ECAR values were normalized to cell number. Basal mitochondrial ATP and glycolytic ATP generation rates were calculated based on OCR and ECAR values using the Seahorse Analytics software.

IV. NMR Spectra

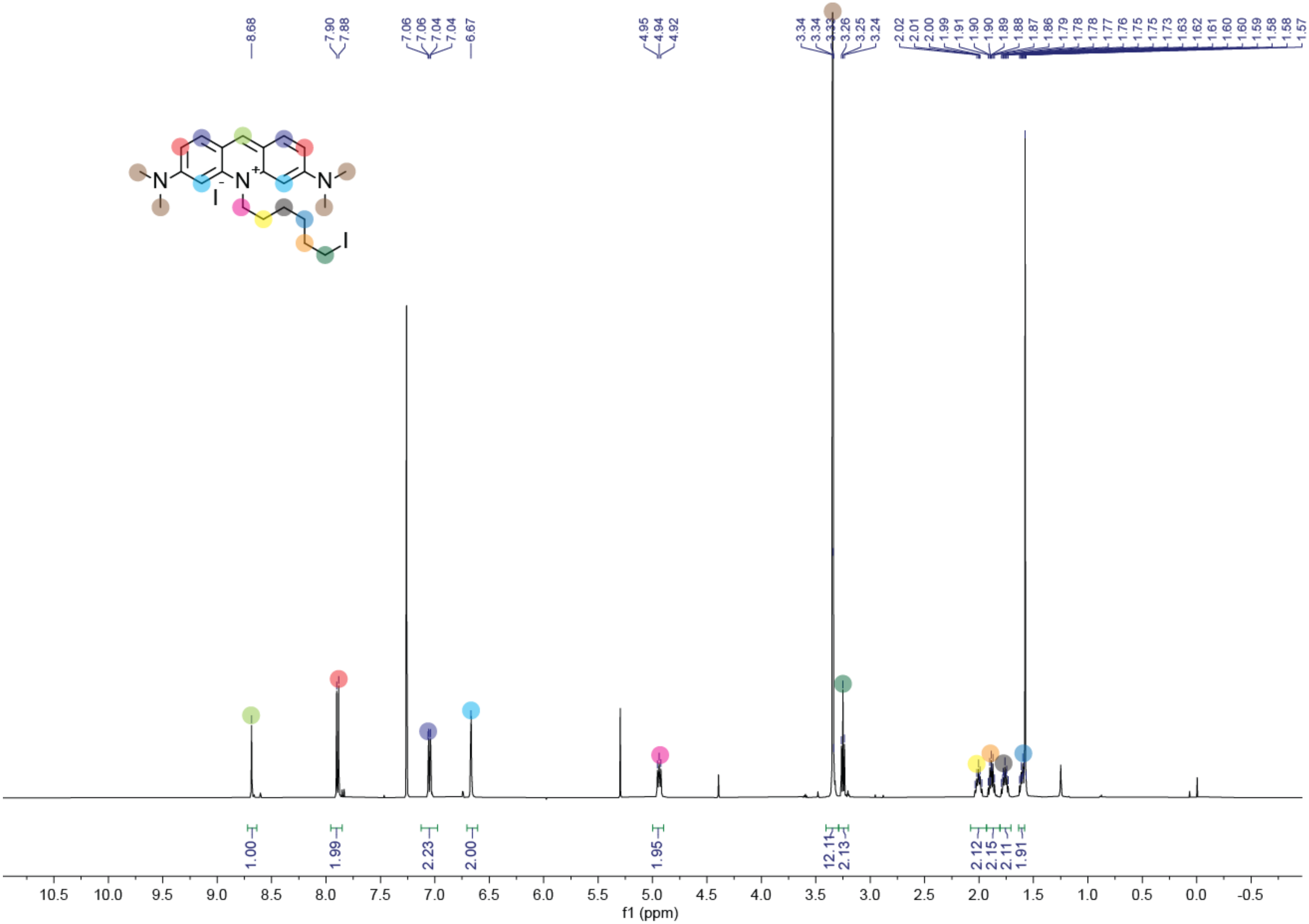
8.68
7.90
7.88
7.06
7.06
7.04
7.04
6.67
4.95
4.94
4.92
3.34
3.34
3.33
3.26
3.25
3.24
2.02
2.01
2.00
1.99
1.91
1.90
1.90
1.89
1.88
1.87
1.86
1.79
1.78
1.78
1.77
1.76
1.75
1.75
1.73
1.63
1.62
1.61
1.60
1.60
1.59
1.58
1.58
1.57
1.00
1.99
2.23
2.00
1.95
12.11
2.13
2.12
2.15
2.11
1.91
f1 (ppm)

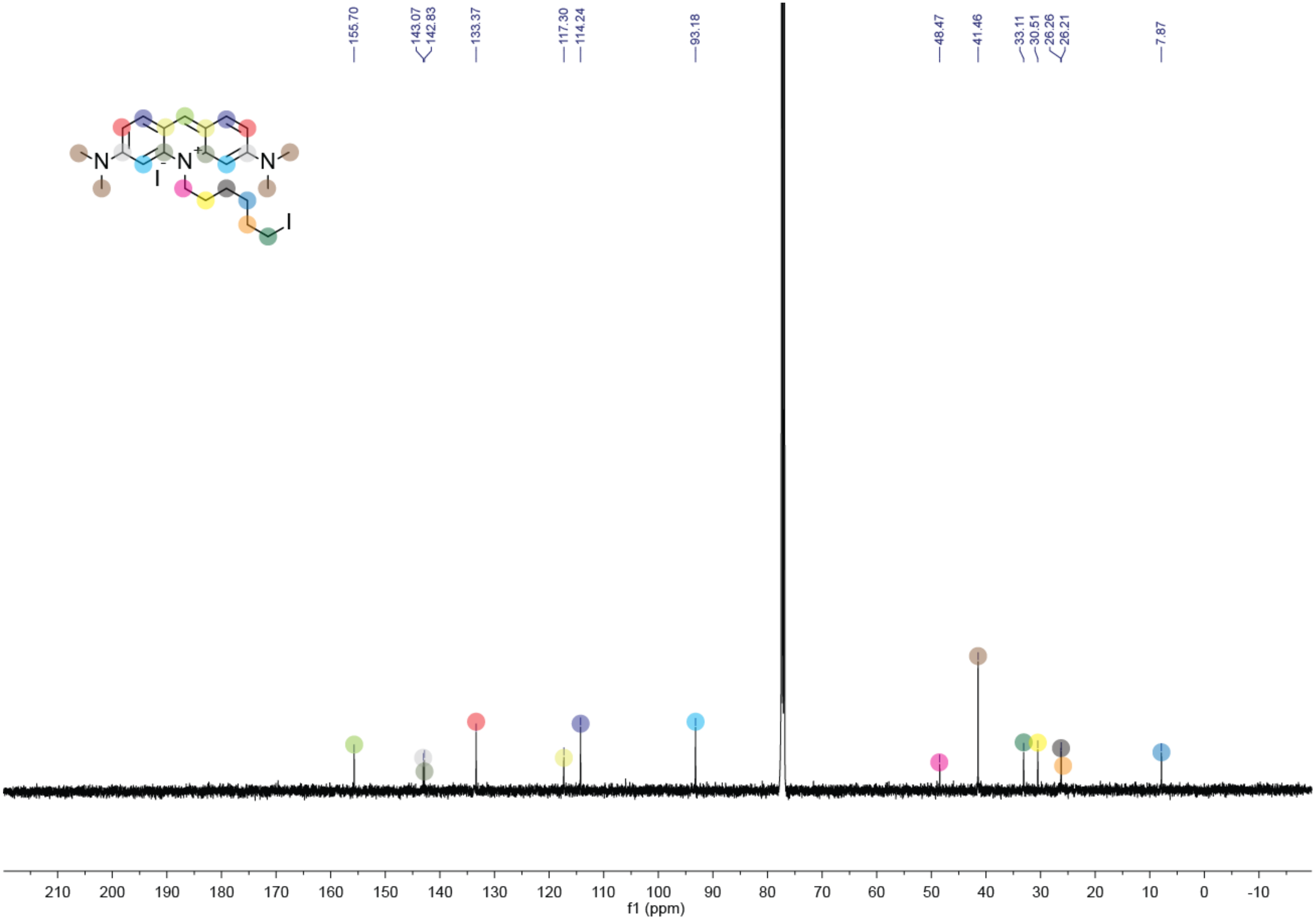
155.70
143.07
142.83
133.37
117.30
114.24
93.18
48.47
41.46
33.11
30.51
26.26
26.21
7.87
N
N+
I-
N
I
210 200 190 180 170 160 150 140 130 120 110 100 90 80 70 60 50 40 30 20 10 0 -10
f1 (ppm)

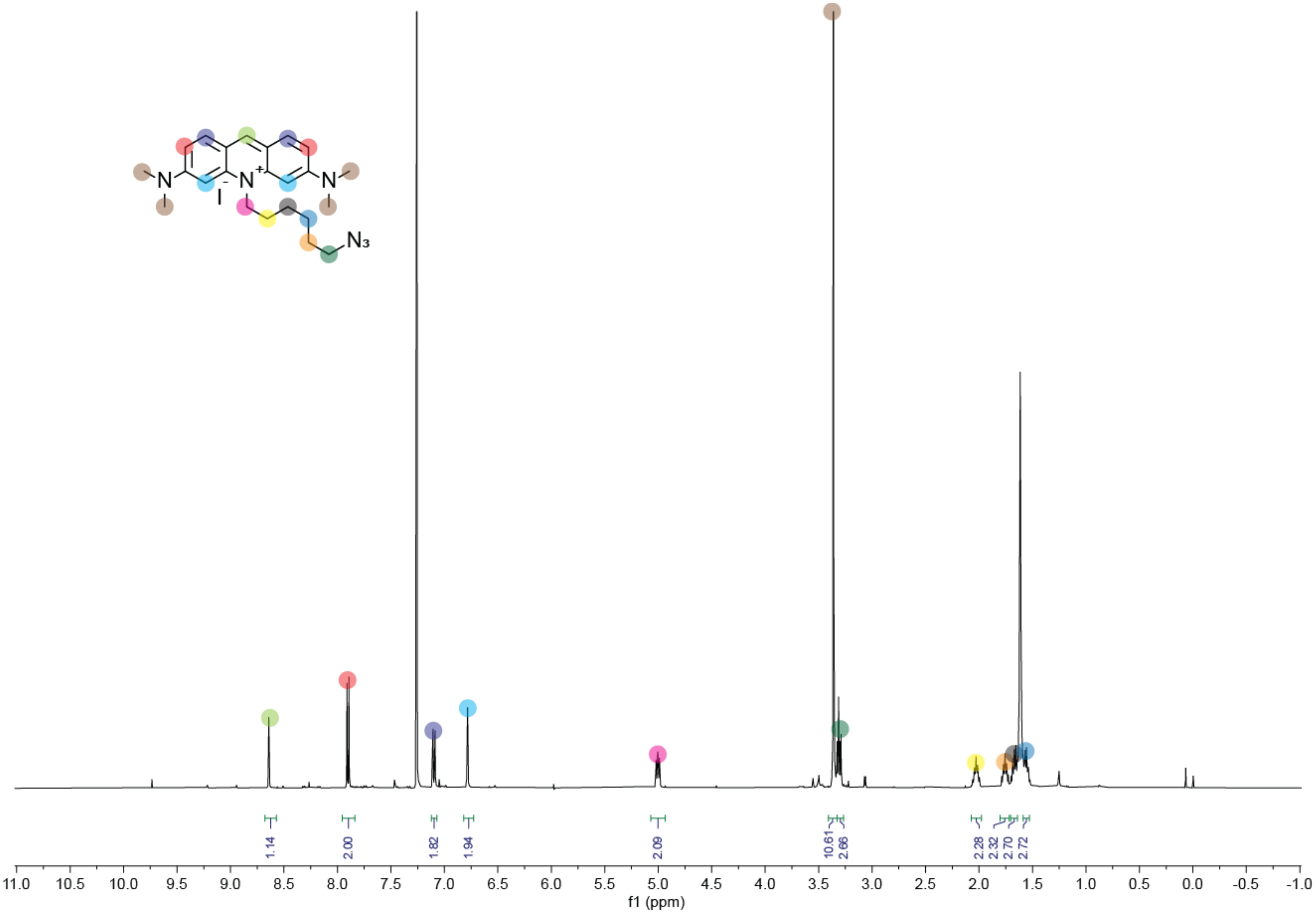

N
N
N
I
N3
1.14
2.00
1.82
1.94
2.09
10.61
2.66
2.28
2.32
2.70
2.72
11.0 10.5 10.0 9.5 9.0 8.5 8.0 7.5 7.0 6.5 6.0 5.5 5.0 4.5 4.0 3.5 3.0 2.5 2.0 1.5 1.0 0.5 0.0 -0.5 -1.0
f1 (ppm)

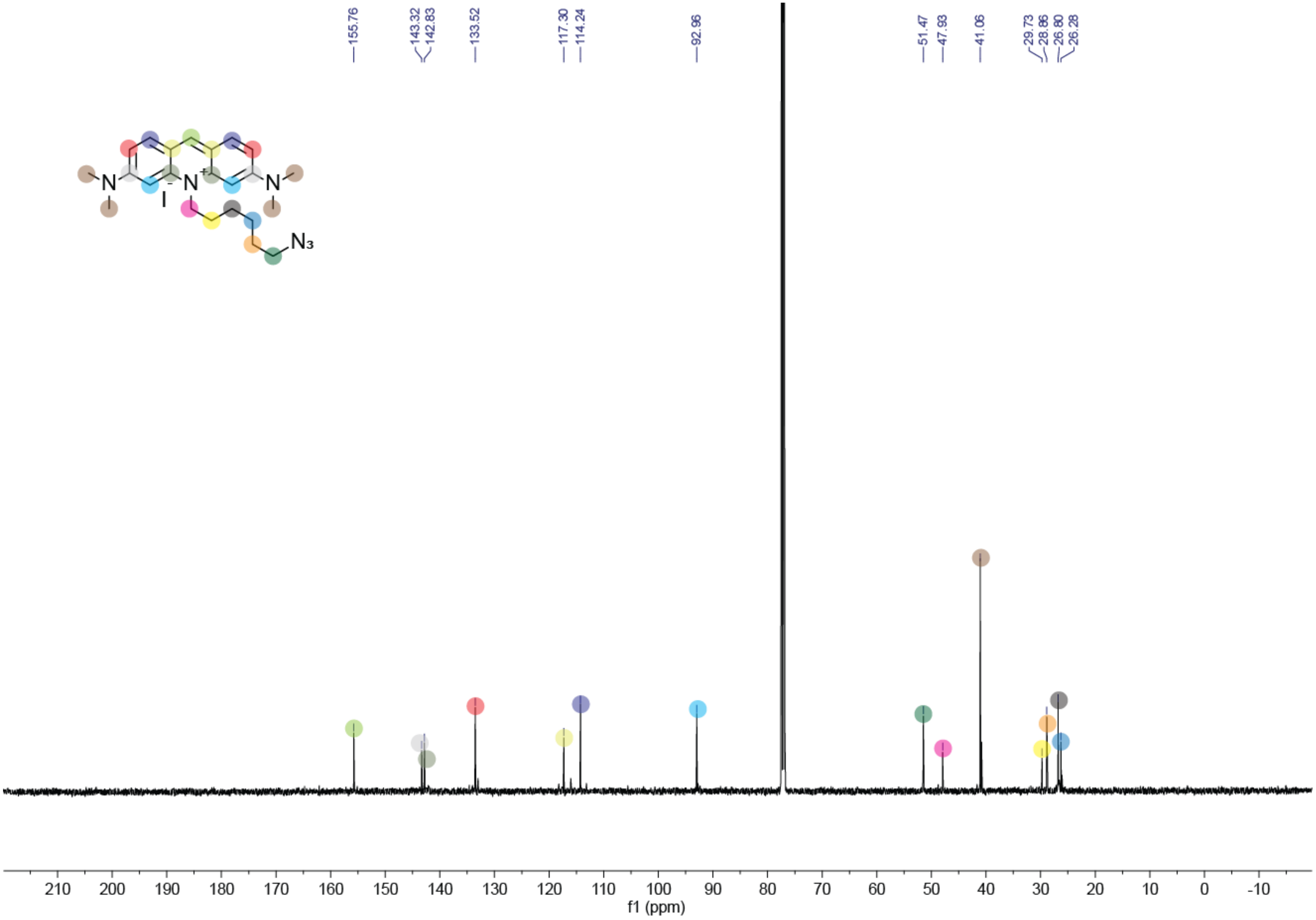

155.76
143.32
142.83
133.52
117.30
114.24
92.96
51.47
47.93
41.06
29.73
28.86
26.80
26.28
N
N+
N
I-
N3
210 200 190 180 170 160 150 140 130 120 110 100 90 80 70 60 50 40 30 20 10 0 -10
f1 (ppm)

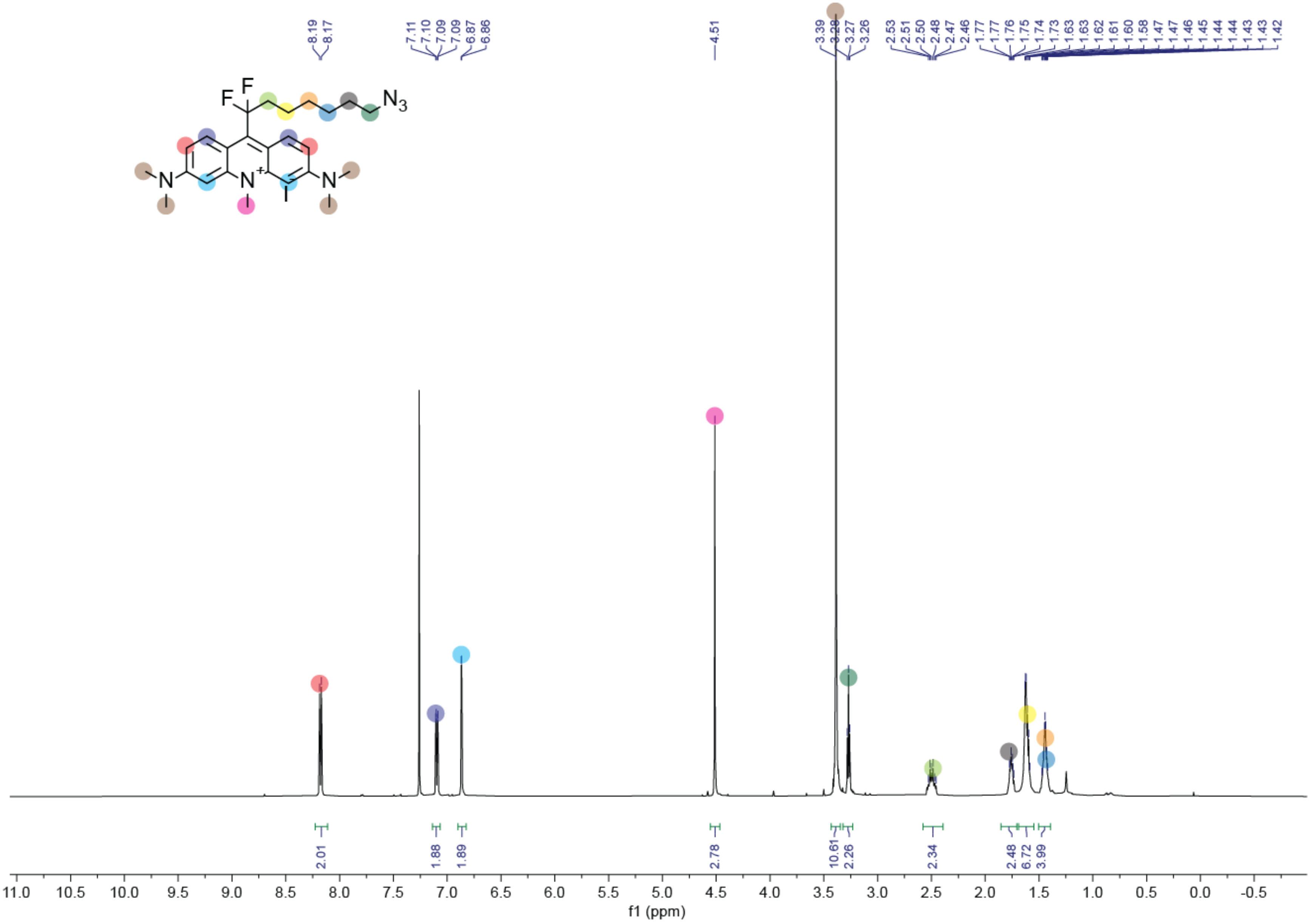
8.19
8.17
7.11
7.10
7.09
7.09
6.87
6.86
4.51
3.39
3.28
3.27
3.26
2.53
2.51
2.50
2.48
2.47
2.46
1.77
1.77
1.76
1.75
1.74
1.73
1.63
1.63
1.62
1.61
1.60
1.58
1.47
1.47
1.46
1.45
1.44
1.44
1.43
1.43
1.42
F
F
N3
N
N+
N
I
2.01
1.88
1.89
2.78
10.61
2.26
2.34
2.48
6.72
3.99
11.0 10.5 10.0 9.5 9.0 8.5 8.0 7.5 7.0 6.5 6.0 5.5 5.0 4.5 4.0 3.5 3.0 2.5 2.0 1.5 1.0 0.5 0.0 -0.5
f1 (ppm)

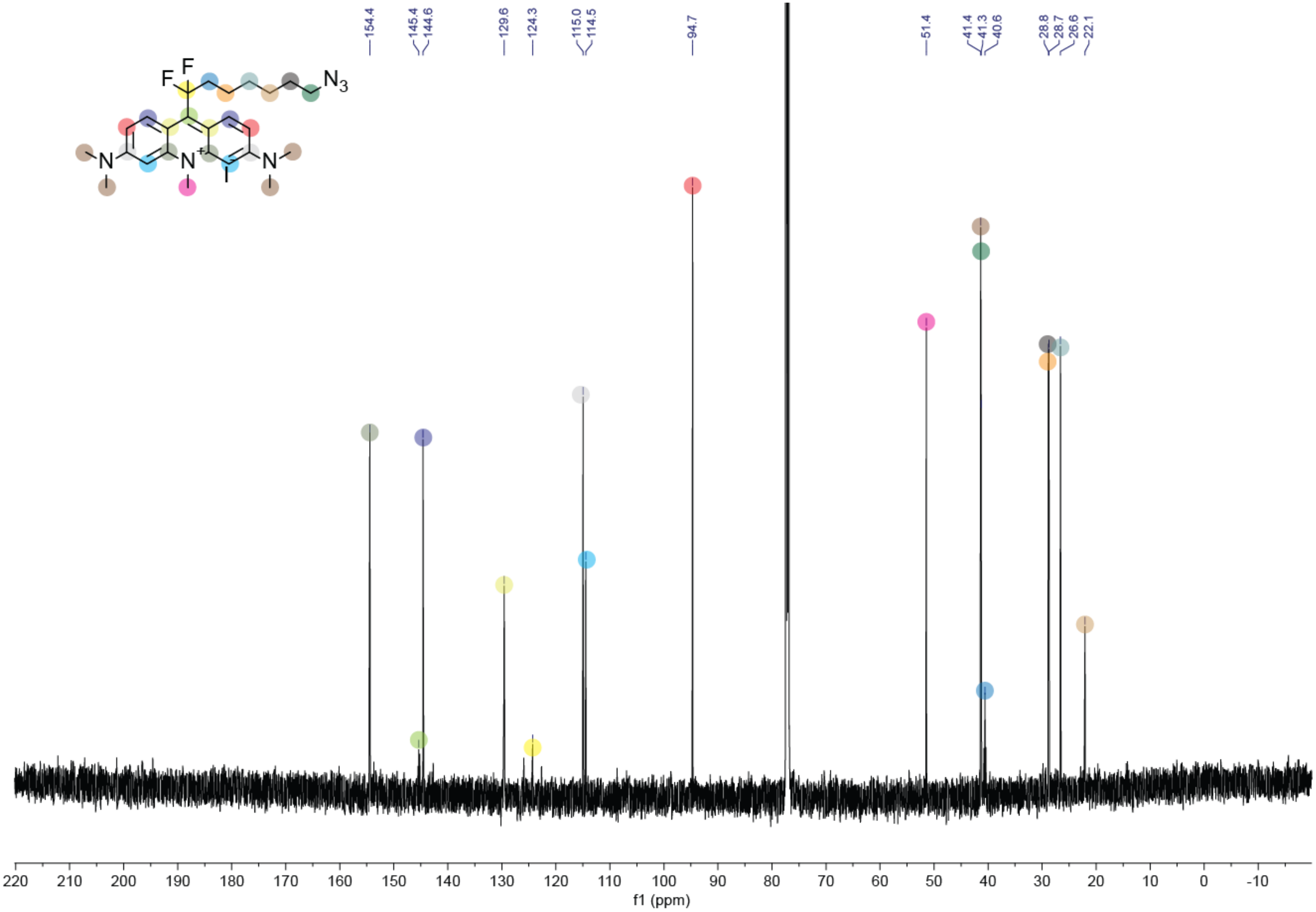
154.4
145.4
144.6
129.6
124.3
115.0
114.5
94.7
51.4
41.4
41.3
40.6
28.8
28.7
26.6
22.1
F
F
N3
N+
N
N
I
220 210 200 190 180 170 160 150 140 130 120 110 100 90 80 70 60 50 40 30 20 10 0 -10
f1 (ppm)

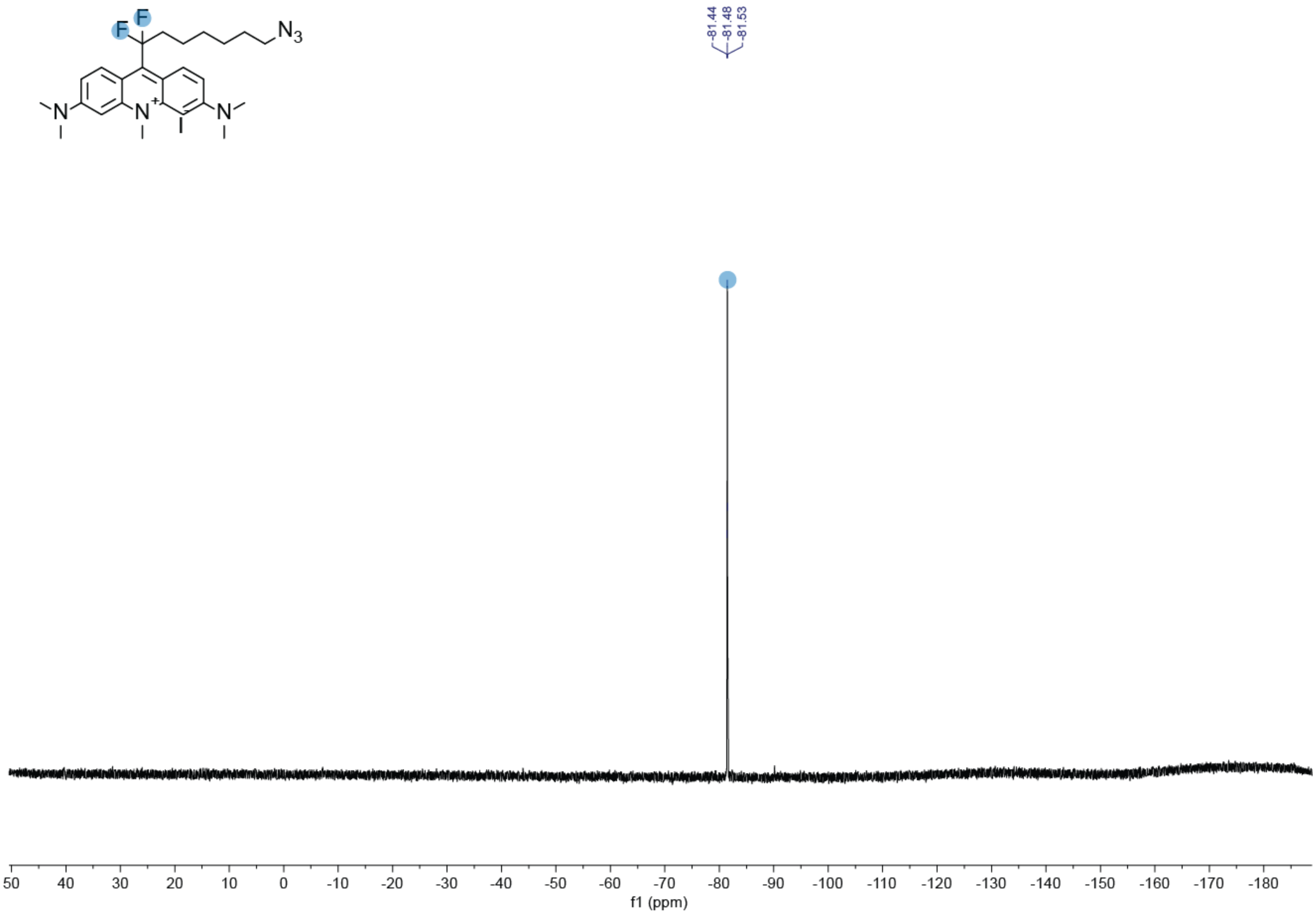
F
F
N3
N
N+
I
N
-81.44
-81.48
-81.53
50 40 30 20 10 0 -10 -20 -30 -40 -50 -60 -70 -80 -90 -100 -110 -120 -130 -140 -150 -160 -170 -180
f1 (ppm)

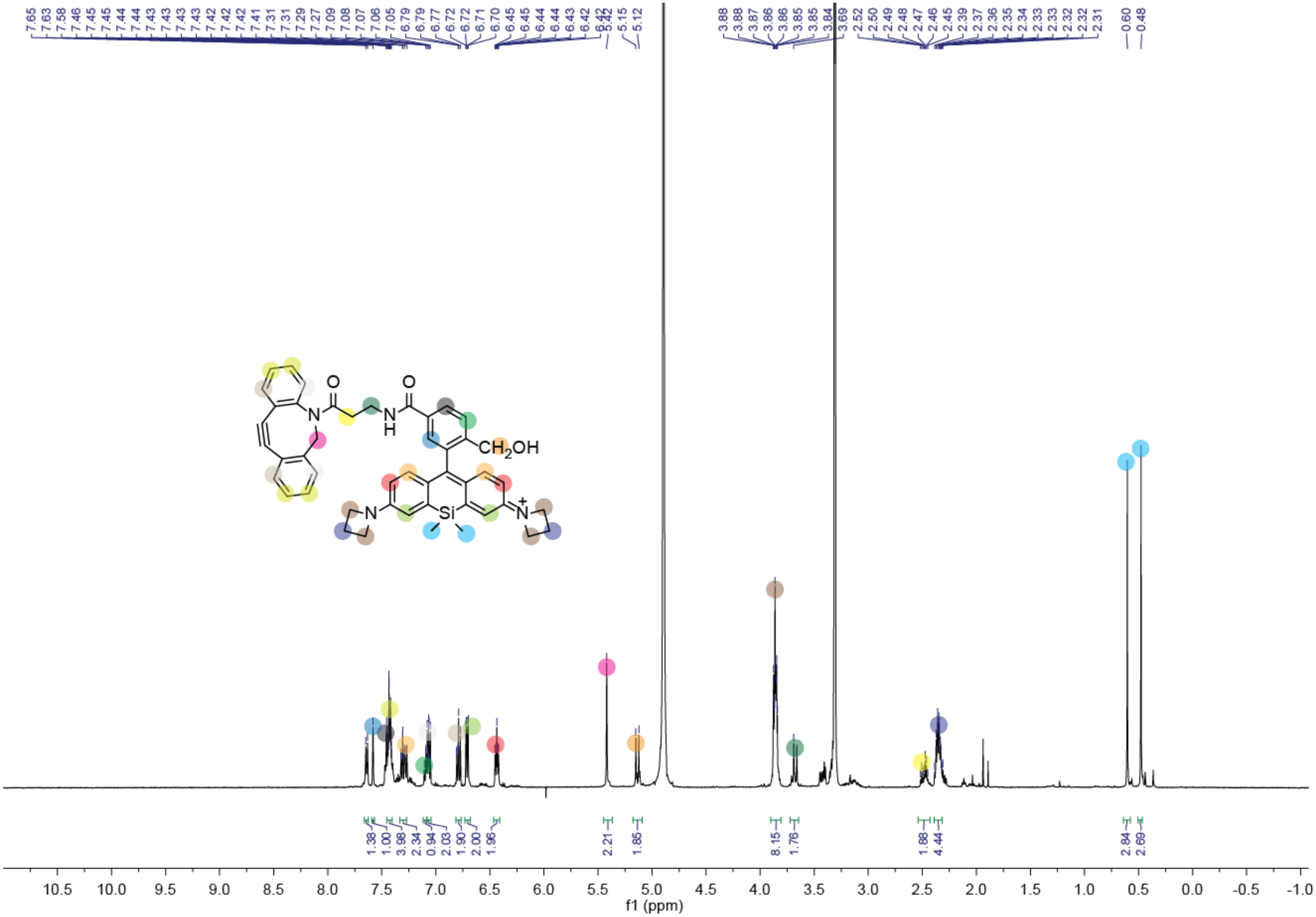
7.65 7.63 7.58 7.46 7.45 7.45 7.44 7.44 7.43 7.43 7.43 7.42 7.42 7.42 7.41 7.31 7.31 7.29 7.27 7.09 7.08 7.07 7.06 7.05 6.79 6.79 6.77 6.72 6.72 6.71 6.70 6.45 6.45 6.44 6.44 6.43 6.42 6.42 5.42 5.15 5.12
3.88 3.88 3.87 3.86 3.86 3.85 3.85 3.84 3.69 2.52 2.50 2.49 2.48 2.47 2.46 2.45 2.39 2.37 2.36 2.35 2.34 2.33 2.33 2.32 2.32 2.31
0.60
0.48
O
N
H
CH2OH
Si
N+
1.38 1.00 3.98 2.34 0.94 2.03 1.90 2.00 1.96 2.21 1.85 8.15 1.76 1.88 4.44 2.84 2.69
10.5 10.0 9.5 9.0 8.5 8.0 7.5 7.0 6.5 6.0 5.5 5.0 4.5 4.0 3.5 3.0 2.5 2.0 1.5 1.0 0.5 0.0 -0.5 -1.0
f1 (ppm)

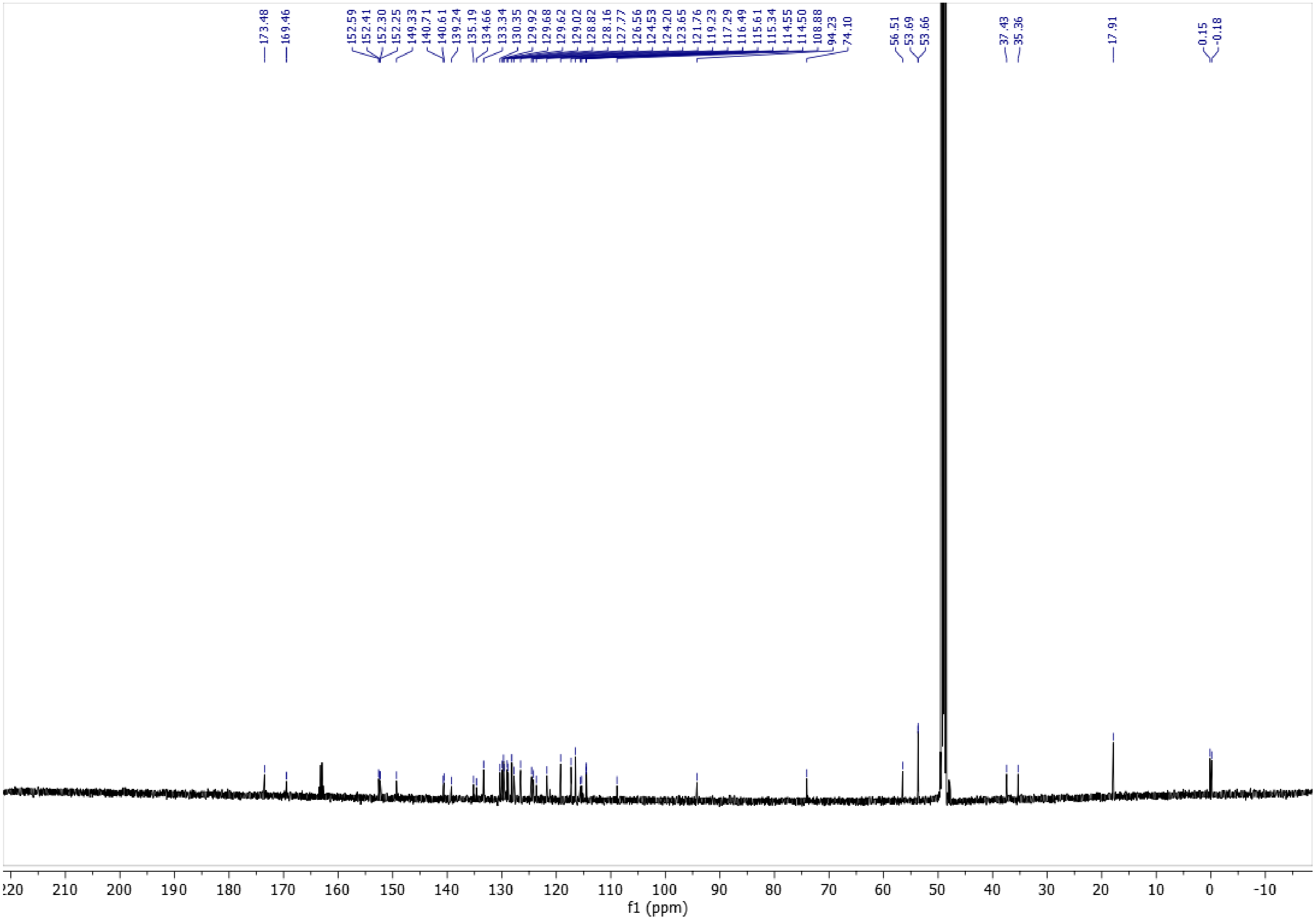
173.48
169.46
152.59
152.41
152.30
152.25
149.33
140.71
140.61
139.24
135.19
134.66
133.34
130.35
129.92
129.68
129.62
129.02
128.82
128.16
127.77
126.56
124.53
124.20
123.65
121.76
119.23
117.29
116.49
115.61
115.34
114.55
114.50
108.88
94.23
74.10
56.51
53.69
53.66
37.43
35.36
17.91
0.15
-0.18
220
210
200
190
180
170
160
150
140
130
120
110
100
90
80
70
60
50
40
30
20
10
0
-10
f1 (ppm)

IV. References

1. Acridine Orange Conjugated Polymersomes for Simultaneous Nuclear Delivery of Gemcitabine and Doxorubicin to Pancreatic Cancer Cells | Bioconjugate Chemistry. https://pubs.acs.org/doi/10.1021/acs.bioconjchem.5b00694.

2. Zhou, Q. *et al.* Bioconjugation by Native Chemical Tagging of C–H Bonds. *J. Am. Chem. Soc.* **135**, 12994–12997 (2013).

3. Chi, W. *et al.* Descriptor ΔGC-O Enables the Quantitative Design of Spontaneously Blinking Rhodamines for Live-Cell Super-Resolution Imaging. *Angew. Chem. Int. Ed.* **59**, 20215–20223 (2020).
