# Supplementary material for: Long-term super-resolution inner mitochondrial membrane imaging with a lipid probe
Source: Nat Chem Biol. 2023 Oct 19;20(1):83–92. doi: 10.1038/s41589-023-01450-y (PMC10746544; doi:10.1038/s41589-023-01450-y)
Supplement: Supplementary file 2 — Reporting Summary [file 41589_2023_1450_MOESM2_ESM.pdf]

nature portfolio

Corresponding author(s): Alanna Schepartz

Last updated by author(s): Jul 14, 2023

Reporting Summary

Nature Portfolio wishes to improve the reproducibility of the work that we publish. This form provides structure for consistency and transparency in reporting. For further information on Nature Portfolio policies, see our Editorial Policies and the Editorial Policy Checklist.

Statistics

For all statistical analyses, confirm that the following items are present in the figure legend, table legend, main text, or Methods section.

n/a	Confirmed	
☐	☒	The exact sample size (*n*) for each experimental group/condition, given as a discrete number and unit of measurement
☐	☒	A statement on whether measurements were taken from distinct samples or whether the same sample was measured repeatedly
☐	☒	The statistical test(s) used AND whether they are one- or two-sided<br>*Only common tests should be described solely by name; describe more complex techniques in the Methods section.*
☐	☒	A description of all covariates tested
☐	☒	A description of any assumptions or corrections, such as tests of normality and adjustment for multiple comparisons
☐	☒	A full description of the statistical parameters including central tendency (e.g. means) or other basic estimates (e.g. regression coefficient) AND variation (e.g. standard deviation) or associated estimates of uncertainty (e.g. confidence intervals)
☐	☒	For null hypothesis testing, the test statistic (e.g. *F*, *t*, *r*) with confidence intervals, effect sizes, degrees of freedom and *P* value noted<br>*Give P values as exact values whenever suitable.*
☒	☐	For Bayesian analysis, information on the choice of priors and Markov chain Monte Carlo settings
☒	☐	For hierarchical and complex designs, identification of the appropriate level for tests and full reporting of outcomes
☒	☐	Estimates of effect sizes (e.g. Cohen's *d*, Pearson's *r*), indicating how they were calculated

Our web collection on statistics for biologists contains articles on many of the points above.

Software and code

Policy information about availability of computer code

Data collection	Leica LAS X 5.1.0, Zeiss Zen Black 2.3, Bruker Topsin 3.6, Waters MassLynx 4.2, Agilent Wave 2.6
Data analysis	SVI Huygens Pro22.04, JaCoP and KymographBuilder on FIJI (ImageJ 2.9.0/1.53t), Mestrelab MestreNova 14.2, Graphpad Prism 8, OriginLab Origin Pro2022, Agilent Seahorse Analytics

For manuscripts utilizing custom algorithms or software that are central to the research but not yet described in published literature, software must be made available to editors and reviewers. We strongly encourage code deposition in a community repository (e.g. GitHub). See the Nature Portfolio guidelines for submitting code & software for further information.

Data

Policy information about availability of data

All manuscripts must include a data availability statement. This statement should provide the following information, where applicable:

- Accession codes, unique identifiers, or web links for publicly available datasets
- A description of any restrictions on data availability
- For clinical datasets or third party data, please ensure that the statement adheres to our policy

The materials and data reported in this study are available upon reasonable request from the corresponding author.

Research involving human participants, their data, or biological material

Policy information about studies with human participants or human data. See also policy information about sex, gender (identity/presentation), and sexual orientation and race, ethnicity and racism.

Reporting on sex and gender	N/A
Reporting on race, ethnicity, or other socially relevant groupings	N/A
Population characteristics	N/A
Recruitment	N/A
Ethics oversight	N/A

Note that full information on the approval of the study protocol must also be provided in the manuscript.

Field-specific reporting

Please select the one below that is the best fit for your research. If you are not sure, read the appropriate sections before making your selection.

☒ Life sciences ☐ Behavioural & social sciences ☐ Ecological, evolutionary & environmental sciences

For a reference copy of the document with all sections, see nature.com/documents/nr-reporting-summary-flat.pdf

Life sciences study design

All studies must disclose on these points even when the disclosure is negative.

Sample size	Sample size was determined using discrete cells exposed to the same conditions, all data was sourced from at least two biological replicates to ensure reproducibility and enable statistical analysis.
Data exclusions	No data was excluded
Replication	All biochemical assay and microscopy experiments were performed with at least three biologically independent replicates. All attempts at replication were successful.
Randomization	Randomization was not performed for biochemical assays and microscopy experiments; randomization is not relevant because samples form defined groups to facilitate statistical comparison.
Blinding	Sample preparation, data collection and analysis was conducted by three individuals (SZ, ND and DM) using standard protocols, investigators were blinded during the data analysis; no difference was shown among individuals.

Reporting for specific materials, systems and methods

We require information from authors about some types of materials, experimental systems and methods used in many studies. Here, indicate whether each material, system or method listed is relevant to your study. If you are not sure if a list item applies to your research, read the appropriate section before selecting a response.

Materials & experimental systems

n/a	Involved in the study	
☒	☐	Antibodies
☐	☒	Eukaryotic cell lines
☒	☐	Palaeontology and archaeology
☒	☐	Animals and other organisms
☒	☐	Clinical data
☒	☐	Dual use research of concern
☒	☐	Plants

Methods

n/a	Involved in the study	
☒	☐	ChIP-seq
☒	☐	Flow cytometry
☒	☐	MRI-based neuroimaging

Eukaryotic cell lines

Policy information about cell lines and Sex and Gender in Research

Cell line source(s)	HeLa cells were obtained from UC Berkeley Cell Culture Facility
Authentication	HeLa cells were authenticated by UC Berkeley Cell Culture Facility using STR profiling; no further authentication was performed
Mycoplasma contamination	Cells were periodically for mycoplasma with DNA models; results were negative.
Commonly misidentified lines (See ICLAC register)	No commonly misidentified lines were used in this study.
