# Supplementary figures and images for: Long-term super-resolution inner mitochondrial membrane imaging with a lipid probe
Source: Nat Chem Biol. 2023 Oct 19;20(1):83–92. doi: 10.1038/s41589-023-01450-y (PMC10746544; doi:10.1038/s41589-023-01450-y)

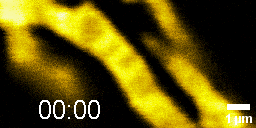

Supplement: Supplementary file 10 — Time-lapse STED imaging of mitochondria labeled with MAO-SiR. HeLa cells were labeled with MAO-N3 and SiR-DBCO (Methods) and imaged using a point-scanning confocal microscope equipped with a 775-nm STED laser (n = 125 frames; t = 162.5 s). [file 41589_2023_1450_MOESM10_ESM.gif]

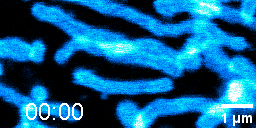

Supplement: Supplementary file 11 — Time-lapse STED imaging of mitochondria labeled with SiR-CA and Halo-TOMM20. HeLa cells expressing the OMM marker Halo-TOMM20 were labeled with SiR-CA (Methods) and imaged using a point-scanning confocal microscope equipped with a 775-nm STED laser (n = 125 frames; t = 162.5 s). [file 41589_2023_1450_MOESM11_ESM.gif]

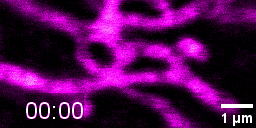

Supplement: Supplementary file 12 — Time-lapse STED imaging of mitochondria labeled with SiR-SNAP and COX8A-SNAP. HeLa cells expressing the OMM marker COX8A-SNAP were labeled with SiR-SNAP (Methods) and imaged using a point-scanning confocal microscope equipped with a 775-nm STED laser (n = 125 frames; t = 162.5 s). [file 41589_2023_1450_MOESM12_ESM.gif]
